# From genetics to biotechnology: Synthetic biology as a flexible course‐embedded research experience

**DOI:** 10.1002/bmb.21662

**Published:** 2022-09-02

**Authors:** Kristen C. Johnson, Jaime L. Sabel, Judith Cole, Christin L. Pruett, Ruth Plymale, Nathan S. Reyna

**Affiliations:** ^1^ Department of Life Sciences University of New Hampshire Manchester New Hampshire USA; ^2^ Department of Biological Sciences University of Memphis Memphis Tennessee USA; ^3^ Department of Biology Ouachita Baptist University Arkadelphia Arkansas USA

**Keywords:** course‐based undergraduate research experience, CURE, synthetic biology

## Abstract

The need for changing how science is taught and the expansion of undergraduate research experiences is essential to foster critical thinking in the Natural Sciences. Most faculty research programs only involve a small number of upper‐level undergraduate students each semester. The course‐based undergraduate research experience (CURE) model enables more students to take ownership over an independent project and experience authentic research. Further, by creating projects that fit into a curriculum's learning goals and student‐oriented outcomes, departments help strengthen critical thinking skills in the classroom. Here, we report on the incorporation of a synthetic biology CURE into a mid‐level cellular biology course and two advanced level genetics/molecular biology courses. Synthetic biology involves systematic engineering of novel organisms, such as bacteria and plants, to work as functional devices to solve problems in medicine, agriculture, and manufacturing. The value of synthetic biology and its ultimate utility as a teaching tool relies on reusable, standard genetic parts that can be interchanged using common genetic engineering principles. This Synthetic biology CURE effectively achieves five essential goals: (1) a sense of project ownership; (2) self‐efficacy: mastery of a manageable number of techniques; (3) increased tolerance for obstacles through challenging research; (4) increased communication skills; and (5) a sense of belonging in a larger scientific community. Based upon our student assessment data, we demonstrate that this course‐based synthetic biology laboratory engages students directly in an authentic research experience and models important elements of collaboration, discovery, iteration, and critical thinking.

## 1 INTRODUCTION

The need for changing how science is taught and the expansion of undergraduate research experience has been demonstrated in the Natural Sciences.^1^, ^2^ The foundation for these changes is the incorporation of course‐based undergraduate research experiences (CUREs).^3^ Most faculty research programs typically involve a small number of upper‐level students each semester; however, the incorporation of research into the classroom can conceivably allow every biology major to participate in experimental research. Further, by creating CUREs that fit into a curriculum's learning goals and student‐oriented outcomes, departments help strengthen critical thinking skills in the classroom. The global improvement of critical thinking skills results in a change in the overall teaching approach to reflect those called upon in the Vision and Change report.^2^ Because of this, many faculty have developed research‐based modules for their classroom. However, these are still only a small portion of what is done during the semester and do not reflect significant research experiences. Effective CUREs act as a complete replacement of the more traditional weekly lab activities.^4^

Here we report on a semester‐long synthetic biology (SynBio) CURE that effectively achieves five essential goals: (1) a sense of project ownership; (2) self‐efficacy: mastery of a manageable number of techniques; (3) increased tolerance for obstacles through challenging research; (4) increased communication skills; and (5) a sense of belonging in a larger scientific community.^5^ The SynBio CURE is based upon access to over 2000 iGEM (International Genetically Engineered Machine) standard BioBrick parts, DNA building blocks that follow a restriction enzyme design assembly standard. Students use BioBricks to design, assemble, and analyze their own functional, unique synthetic genetic devices. Student work throughout the synthetic biology CURE requires learning basic recombinant DNA cloning techniques, which are iterative in nature, combined with careful experimental design and statistical analysis. Positive and negative controls, reliability, and reproducibility are critical aspects of these experiments. Critical thinking through obstacles builds students' toolkits for approaching research problems. Clear documentation in lab notebooks, communication with team members, and class presentations of methods and data further promote student skills in problem‐solving and their sense of project ownership. Students routinely interact with the online iGEM community throughout their project in order to obtain information about their BioBrick parts, allowing them to see the significance of their project and to feel a sense of community.^6^ By researching, designing and then engineering their own devices in the lab, students experience the thrill of research—discovering something that had been completely unknown before. Throughout this lab, students can see how a single mutation or external manipulation results in numerous changes in gene expression. This allows students to comprehend gene expression in a way no lecture or demonstration can communicate. The exhilaration of discovery is highly motivating for students and teachers alike and serves to stimulate continued interest in research.

Synthetic biology involves systematic engineering of novel organisms, such as bacteria and plants, to work as functional devices to solve problems in medicine, agriculture, and manufacturing. The value of synthetic biology relies on reusable, standard genetic parts that can be interchanged using common engineering principles.^7^ Because of this, assembly of novel biological systems derives from a methodical process. Current examples of the use of synthetic biology in research applications range from engineering bacteria as biosensors for water contamination to the production of anti‐malaria drugs and human insulin to reprogramming bacterial genomes.^8^, ^9^, ^10^ Because synthetic biology is multi‐dimensional and borrows principles from many different disciplines,^7^ it allows for integration of diverse techniques and ideas into the classroom and the lab. Synthetic biology promotes the incorporation of bioinformatics in the classroom and serves as a bridge between in silico identification of putative promoters, terminators, and ribosome binding sequences and the wet lab experience.^11^, ^12^, ^13^ The methodical nature of synthetic biology assembly, which utilizes standard genetic parts, lends itself well to learning cloning through iterative processes in undergraduate research‐focused courses.

## 2 COURSE ORGANIZATION AND ASSESSMENT

### 2.1 Faculty training and implementation

The Arkansas (AR) CURE project supported a 2.5 day workshop, hosted at Ouachita Baptist University (Arkadelphia, AR) to help faculty implement the SynBio CURE at their home institutions. Through this training, AR‐CURE provided faculty with a “turn‐key” semester‐long CURE in synthetic biology. From 2017 to 2020, 87 faculty from 75 institutions, comprising community colleges, tribal colleges, primarily undergraduate institutions (PUIs) and research‐oriented universities across 30 states, attended the AR‐CURE SynBio training (Figure S1). During the training, faculty attend workshops to learn how to implement the lab course materials at their home institutions and are provided with lab protocols and helpful tips from experienced SynBio faculty.

An open‐source online lab manual is now available through LibreText (https://bio.libretexts.org/Bookshelves/Biotechnology/Lab_Manual%3A_Synthetic_Biology_Protocols), which includes student protocols and instructor notes, including recipes and lab set‐up directions. A series of training videos have been created to help faculty incorporate the SynBio lab into their curriculum. Videos include information on lab design and organization, pedagogy tips and assessment, and grading lab activities. Faculty interviews have been recorded to provide a range of implementation examples and strategies. The full SynBio video playlist is available on YouTube (https://youtube.com/playlist?list=PLVl417rgx4ahv‐p7ecFQJ43mvZIoSczKk).

The flexibility of the SynBio CURE has led to its successful implementation across a variety of institutions. However, for continuity, in this article, we highlight a model for the SynBio CURE and report student outcomes from advanced level genetics/molecular biology courses with enrollments of 20–40 students at two institutions (Ouachita Baptist University [AR], the University of New Hampshire‐Manchester [NH], and a mid‐level cellular biology course at the University of Memphis [TN]). At each institution, the lab is similar with only minor institutional specific differences (Table S1).

**FIGURE 1:**
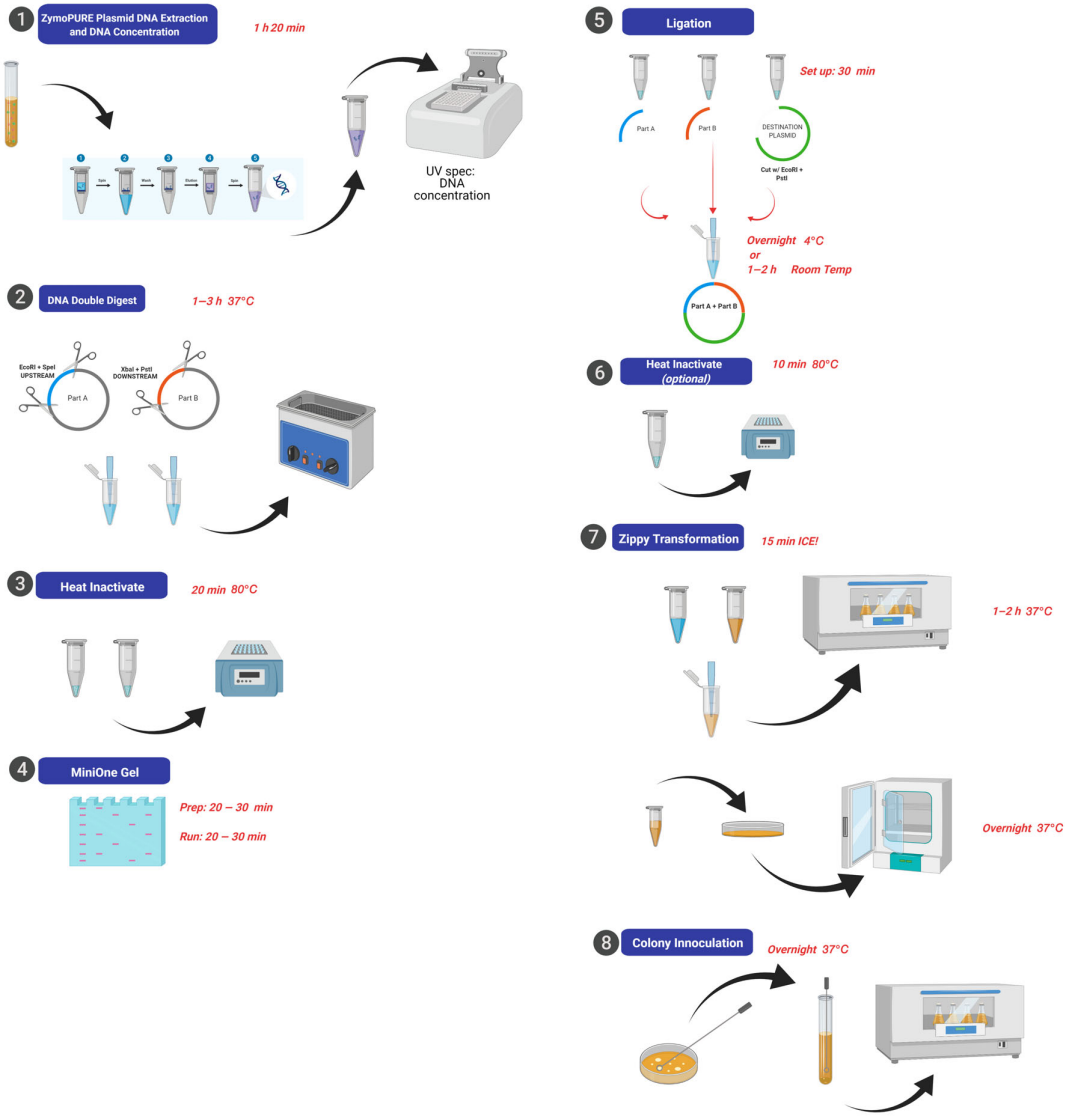
Cloning process for BioBrick device assembly. Protocols for each step provided in the online lab manual (LibreText). Temperature for incubation and estimated time for each step indicated (in red). Created with BioRender.com

The SynBio lab, as described, is intended as a CURE to accompany a semester‐long advanced molecular biology/genetics course for biology majors. The lab is designed to fit into one 3‐h lab period with some additional outside class time required (inoculate broths, remove plates from incubator) or for lab meetings 2 h twice per week with no outside class time required. Lab protocols were developed that help students and instructors identify time requirements and stopping points (Figure 1). However, the flexibility of this CURE allows for faculty to develop a modular project and tailor the lab to institutional needs and course level. For example, the University of Memphis CURE has modified this model to engage mid‐level cellular biology students who meet for 3 h two times per week.

### 2.2 Background

**FIGURE 2:**
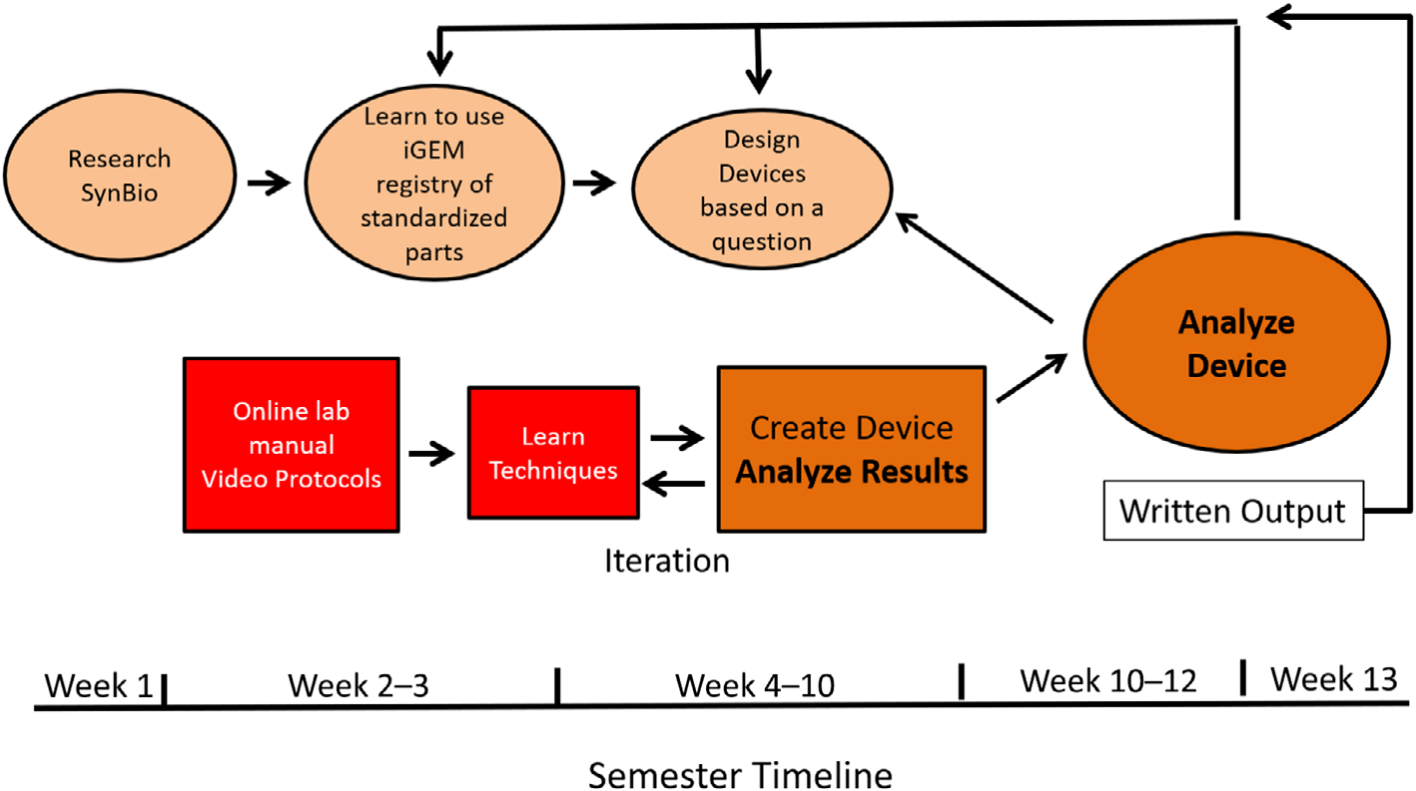
SynBio CURE course workflow. Introductory material and project design is presented during the first 3 weeks of the SynBio lab CURE, followed by ample time for student research, final data analysis and project presentations

To grasp the content in the SynBio CURE labs, accompanying instruction in the areas of prokaryotic gene structure and gene expression regulation is important. The understanding of promoter structure, ribosome binding sites/sequences, coding sequences and reporter genes, and transcriptional terminators are critical to the design phase of this lab. Additionally, cloning is a critical component of this course, and students should be taught basic recombinant DNA vocabularies, such as plasmids, restriction enzymes, ligase, gel electrophoresis, and bacterial transformation. As implemented at the three institutions in this manuscript, these concepts are introduced in accompanying lectures and reinforced in lab activities. In order to facilitate the SynBio CURE, we have compiled an online lab manual that consists of written and video protocols (https://bio.libretexts.org/Bookshelves/Biotechnology/Lab_Manual%3A_Synthetic_Biology_Protocols), a semester outline (Figure 2), as well as ancillary materials that can be utilized to help students understand the concept of BioBricks and assembling a device (File S1).

### 2.3 Student learning objectives

We created learning objectives for the SynBio CURE according to standard methodologies for writing educational objectives.^14^, ^15^

At the end of the SynBio CURE, students will be able to:

1. Keep and maintain a research lab notebook.
2. Create and deliver a scientific presentation.
3. Successfully, perform techniques associated with molecular cloning.
4. Evaluate and troubleshoot molecular cloning techniques to ensure optimum performance.
5. Describe the role that promoter sequences, ribosome binding sequences, and transcription terminators play in regulating gene expression.
6. Design a functional synthetic device using BioBricks.
7. Design experiments to prove the creation of a functional device and evaluate the device's efficiency associated with gene expression.
8. Evaluate their own experimental data.
9. Explain the ethical and social implication of emerging technologies associated with synthetic biology and genetics.
10. Develop critical thinking as the result of participation in the course.

### 2.4 Course structure

**FIGURE 3:**
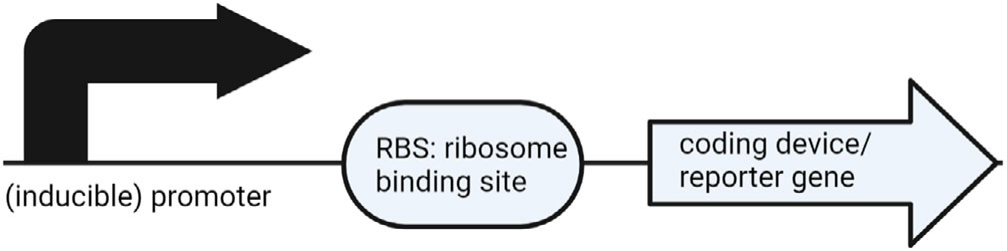
Basic SynBio project device design. In this basic biosensor design, an inducible promoter is attached to a ribosome binding site (RBS) and a reporter gene. Created with BioRender.com

Conceptually, the SynBio CURE encourages students to design a synthetic device that can be expressed in a bacterium to address a biological question. Essentially, students design their own *E. coli*‐based biosensor (e.g., a biosensor that will detect soil pH). These devices typically contain, at their most basic level, an inducible promoter, a ribosome binding site, and a reporter gene (Figure 3). To understand the concepts of synthetic biology and learn how to create their device, students are introduced to this area of research and the tools for cloning in a series of introductory lessons during Weeks 1–3 of the course (Figure 2). Student research proceeds during Weeks 4–12 of the course in which students clone and analyze their device. All student notes and data are recorded in a lab notebook. The iterative cloning process results in mastery of many molecular biology techniques and enables consistent opportunities for problem solving (Table 1).

**TABLE 1:** Techniques students are expected to master in the SynBio CURE

Micro pipetting
Plasmid cloning: type II restriction enzyme digests and ligations
Plasmid DNA mini prep
DNA quantitation using UV spectroscopy
Gel electrophoresis
Bacterial transformation
Culturing *E. coli* in solid and liquid media

Week 1: Faculty provide basic background on synthetic biology as distinct from genetic engineering due to the use of standardized parts. Through the use of examples in the literature, such as Saeidi et al, *Engineered microbes to sense and eradicate Pseudomonas aeruginosa, a human pathogen* ^16^ and through self‐guided investigations of prior iGEM projects utilizing the iGEM website (igem.org), students are exposed to real‐world applications of synthetic biology. Working in small teams, students research and explore several synthetic biology‐based projects on the iGEM website and learn about the standardized nature and modularity of the BioBrick parts that are utilized to build systems, such as biosensors. Students then report their findings to the rest of the class in an effort to build a broad appreciation for synthetic biology designs.

Week 2: A focus on techniques allows students to learn basic molecular biology skills (if needed), such as micro pipetting, use of UV spectrophotometer to obtain and calculate DNA concentrations, and so forth. Additionally, students learn about how to access available BioBrick parts on the iGEM registry through a student “search and find” spreadsheet activity (File S1).

**FIGURE 4:**
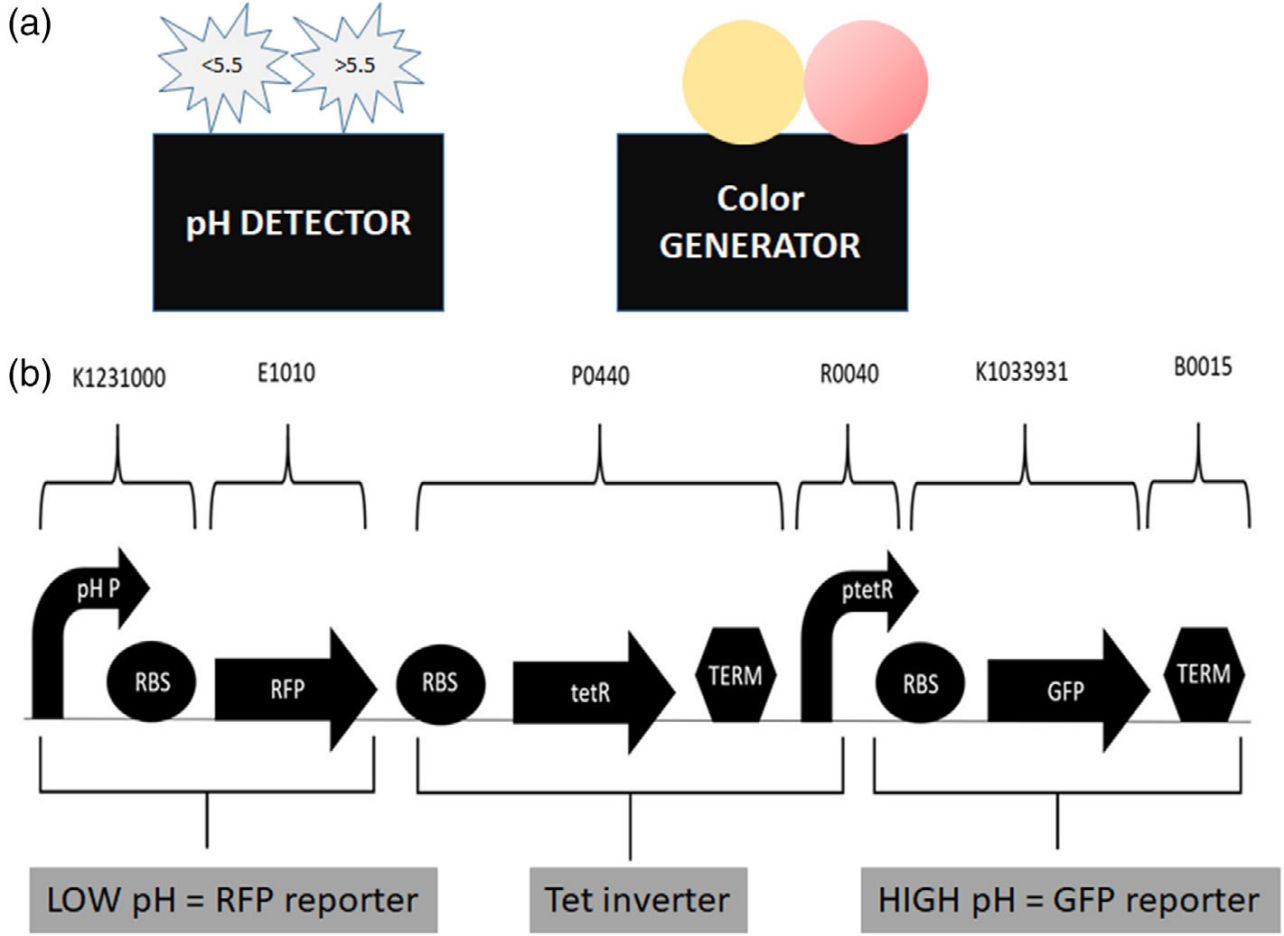
(a) Student project abstraction. Simplified abstraction of synthetic biology device design, displaying a system that detects pH and has a color generator as output. (b) Example student parts level design. Individual BioBrick parts that make up the device are indicated with standard symbols, part names, and part numbers (brackets). The function of combined BioBrick parts is indicated in the boxes below the parts. In this case, this completed device will fluoresce red under low pH conditions and fluoresce green under high pH conditions due to the incorporation of a pH‐sensitive promoter.

Week 3: Students outline a proposal for engineering their own device utilizing BioBricks derived from the iGEM registry of standard parts. Student proposals will initially utilize abstraction (Figure 4a) to model their device for a lay audience and then work toward a detailed parts design (Figure 4b).^7^ A proposal presentation template is provided (File S1). To create a simple device, students may choose at least one promoter, one ribosome binding site (RBS), and one coding sequence that they will clone together in a stepwise process referred to as 3A cloning. However, student teams are encouraged to utilize available composite biobrick parts (e.g., a promoter already linked to an RBS) in order to facilitate easier assembly and the building more complex devices. It is imperative at this stage that students work carefully to design a step‐wise cloning strategy that they can implement to assemble their device.

**FIGURE 5:**
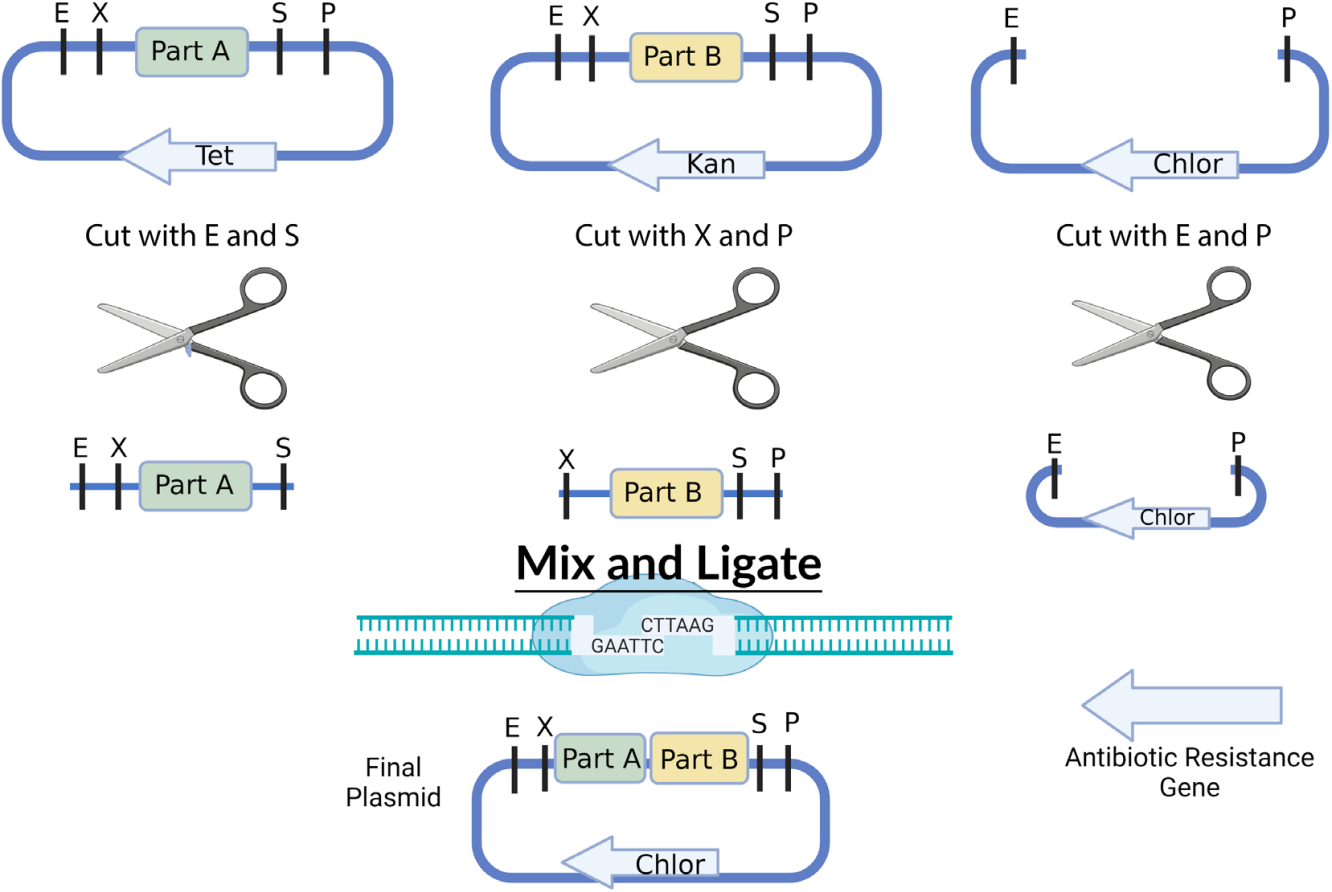
3A assembly process. BioBrick parts are digested out of their backbone plasmids using restriction enzyme combinations that allow them to be ligated together into a third (destination) plasmid that maintains the prefix, E (EcoRI) and X (XbaI), and suffix, S (Sal I) and P (PstI), sequences. This is due to the annealing of the complementary overhangs of SalI and XbaI that result in a merged restriction site no longer recognizable by either enzyme. It is important to note that in order to achieve successful 3A cloning, the destination plasmid must have a different antibiotic resistance marker than either of the plasmids from which BioBrick parts have been digested. Created with BioRender.com

Alongside their project design, students learn about the iterative cloning process utilized to create their device. The nuts and bolts of 3A assembly (Figure 5) are taught through a brief PowerPoint presentation and supported through a very effective hands‐on paper‐cutting and pasting activity (File S1). The 3A cloning process utilizes just four restriction enzymes (EcoRI, PstI, SpeI, and XbaI) and four plasmid backbones. BioBrick pieces are cut two at a time out of their plasmid backbone (e.g., Part A with EcoRI and SpeI; Part B with XbaI, and PstI) and ligated into a new linearized plasmid backbone (cut with EcoRI and PstI) containing a different antibiotic resistance marker (chloramphenicol, ampicillin, kanamycin, and tetracycline). The DNA overhangs of SpeI and XbaI overlap and anneal during the ligation; however, these result in a merged (“M”) site between the two BioBricks that is no longer cut by either restriction enzyme following the ligation. Each cloning step results in a new/fused BioBrick part in which the prefix (EcoRI, XbaI) and suffix (SpeI, PstI) restriction enzyme sites are retained for another round of cloning. This stepwise process is repeated until the student's entire device is cloned. The iterative nature of 3A assembly, as illustrated in Figure 5, allows students to master cloning techniques throughout the semester and to readily problem solve when steps go awry.

Weeks 4–10: Students utilize standard cloning protocols following an online lab manual to assemble their devices. (Figure 1; LibreText). Throughout the research process, students are scheduled to give updates on their project progress through lab meeting presentations or submitted lab report updates. This allows students to practice their scientific presentation skills as well as give feedback to one another, at times recognizing common sources of experimental error or difficulties with techniques.

Weeks 10–12: Students analyze their devices. Once assembled, students will follow through with steps they have proposed to analyze the function of their device. Because each student project is unique and each project cloning timeline may be different, the final stage of the project may incorporate a range of qualitative and quantitative techniques. Students are encouraged to seek out evidence from prior publications and/or prior iGEM team projects in learning about the best modes for analysis.

Weeks 13–15: Students conclude their research project by presenting their device design and data analysis through oral and/or poster presentations and written lab reports. Once completed, students submit their newly created devices as well as their analysis and results to the iGEM website to share with the broader scientific community.

### 2.5 Experimental procedure

The device cloning steps and other experimental procedures are carried out by students according to their device design. Once students have identified the BioBrick parts they require for their project design, these are procured from the appropriate location in an iGEM kit, provided to the student, and they begin the process of transformation and DNA plasmid preparation (steps 7, 8, 1; Figure 1) prior to digestion of their BioBrick parts for ligation. Full experimental protocols are provided in the open‐source LibreText online lab manual (https://bio.libretexts.org/Bookshelves/Biotechnology/Lab_Manual%3A_Synthetic_Biology_Protocols).

### 2.6 Assessment of student learning

Assessment of student learning is divided into three sections: (1) group research (project outlines/proposals, project update presentations, notebook checks), (2) individual knowledge, and (3) final group research results. Each section is worth approximately the same percentage of the overall lab grade. At the beginning of the lab, students must complete weekly individual assignments intended to familiarize them with aspects of synthetic biology. Research groups must turn in outlines/proposals that serve as a research plan for each project. Students are not allowed to begin their projects until the plan is approved. Research outlines are continually updated and periodically graded throughout the semester. Students are required to keep a research notebook. Research notebooks are assessed throughout the semester and at the end of the semester. Student groups are required to give a series of research presentations (project updates) throughout the semester. The final research grade is evaluated based upon a final research presentation, final notebook assessment, and a written project analysis report.

Examples of assignments, rubrics, and representative student proposal and student final presentation are provided in Files S1 and S2.

## 3 EVIDENCE OF STUDENT LEARNING

We utilized several surveys to assess the efficacy of the SynBio CURE with students across multiple universities from 2018 to 2021. Here, we report on the results of two of the surveys as evidence of student learning in the SynBio CURE. The Laboratory Course Assessment Survey (LCAS) examines students' perceptions of biology lab courses and evaluates three‐course design features: (1) collaboration, (2) discovery and relevance, and (3) iteration.^17^ As discussed by Corwin and colleagues (2015), the LCAS may be used to assess the degree to which collaboration, discovery and relevance, and iteration are present in a particular course. The Persistence in the Sciences (PITS) Survey is designed to capture curricular changes that promote undergraduate persistence in science, technology, engineering, and mathematics (STEM) disciplines.^18^ This survey incorporates a six‐factor structure consisting of project ownership (emotion and content), self‐efficacy, science identity, scientific community values, and networking. The LCAS and PITS survey have been validated as useful measures for assessing CURE learning objectives.^17^, ^18^ Cronbach's alpha values,^19^ based on surveys of students taking the Synthetic Biology CURE (*α* ≥ 0.68; Table 2), show that the LCAS and PITS survey queried items have internal consistency and thus are reliable instruments for measuring student attitudes and persistence in the sciences, as was found by Corwin et al. (2015) and Hanauer et al. (2016). All surveys were carried out corresponding to standards established through University Institutional Review Boards according to the following approved protocols: IRB#7023 (UNH); IRB#REY120618 (OBU); IRB#PRO‐FY2019‐83 (UMemphis).

**TABLE 2:** Summary statistics based on PITS and LCAS survey scores for students who had taken a synthetic biology CURE laboratory

Survey	Mean	SD	Cronbach's *α*
PITS *(all out of* 5*)*			
Project ownership‐content	3.62	1.18	0.90
Project ownership‐emotion	3.35	0.99	0.89
Self‐efficacy	4.40	0.68	0.83
Self‐identity	4.07	0.99	0.87
Scientific community values	4.35	0.86	0.83
Networking	3.62	1.29	0.81
LCAS			
Collaboration *(out of* 4*)*	3.50	0.88	0.68
Discovery and relevance *(out of* 5*)*	4.26	0.78	0.85
Iteration *(out of* 5*)*	4.15	0.85	0.78

*Note*: The mean and SD of the students' answers were determined by converting the Likert scale to a numerical scale where Strongly Agree = 5, Agree = 4, Neither agree nor disagree = 3, Disagree = 2, and Strongly Disagree = 1 for all survey questions, except for LCAS‐Collaboration. LCAS‐Collaboration questions had a four‐point scale with 1 = never; 2 = once or twice; 3 = monthly; 4 = weekly. Cronbach's (1951) alpha was measured using the package “ltm”^20^ in R^21^ to ensure the reliability or internal consistency of the PITS and LCAS test items. All scores were out of 5, except for LCAS‐Collaboration (out of 4).

### 3.1 Evidence of achievement of CURE goals

A good CURE, similar to a good independent research project, encourages students to collaborate with others, have a sense of discovery, and allows for students to revise procedures.^5^, ^22^, ^23^ The LCAS was our first survey approved by the IRB and as a result was administered to more students than the PITS survey. We utilized all of the questions from the published LCAS, a post‐survey, to assess levels of scientific collaboration, discovery and relevance, and experimental iteration^17^ for 132 students at five institutions from the spring of 2018 to the spring of 2021. Of note, our survey employed a five‐point Likert scale for all questions in the discovery and relevance and iteration sections compared with Corwin and colleagues published work in 2015 which used a 6‐point scale for these questions.

Ninety‐four percent of the 132 students surveyed were upper‐level undergraduates (Junior or Senior). While we did not collect demographic information for this survey, we can report that 91% of the students were Biology (60%), Biotechnology (18%), or Biomedicine (13%) majors. The SynBio CURE was the first research experience of any type for 35% of the participating students. Interestingly, 21% of the students indicated they had participated in an independent research project but that the SynBio lab was their first CURE.

**FIGURE 6:**
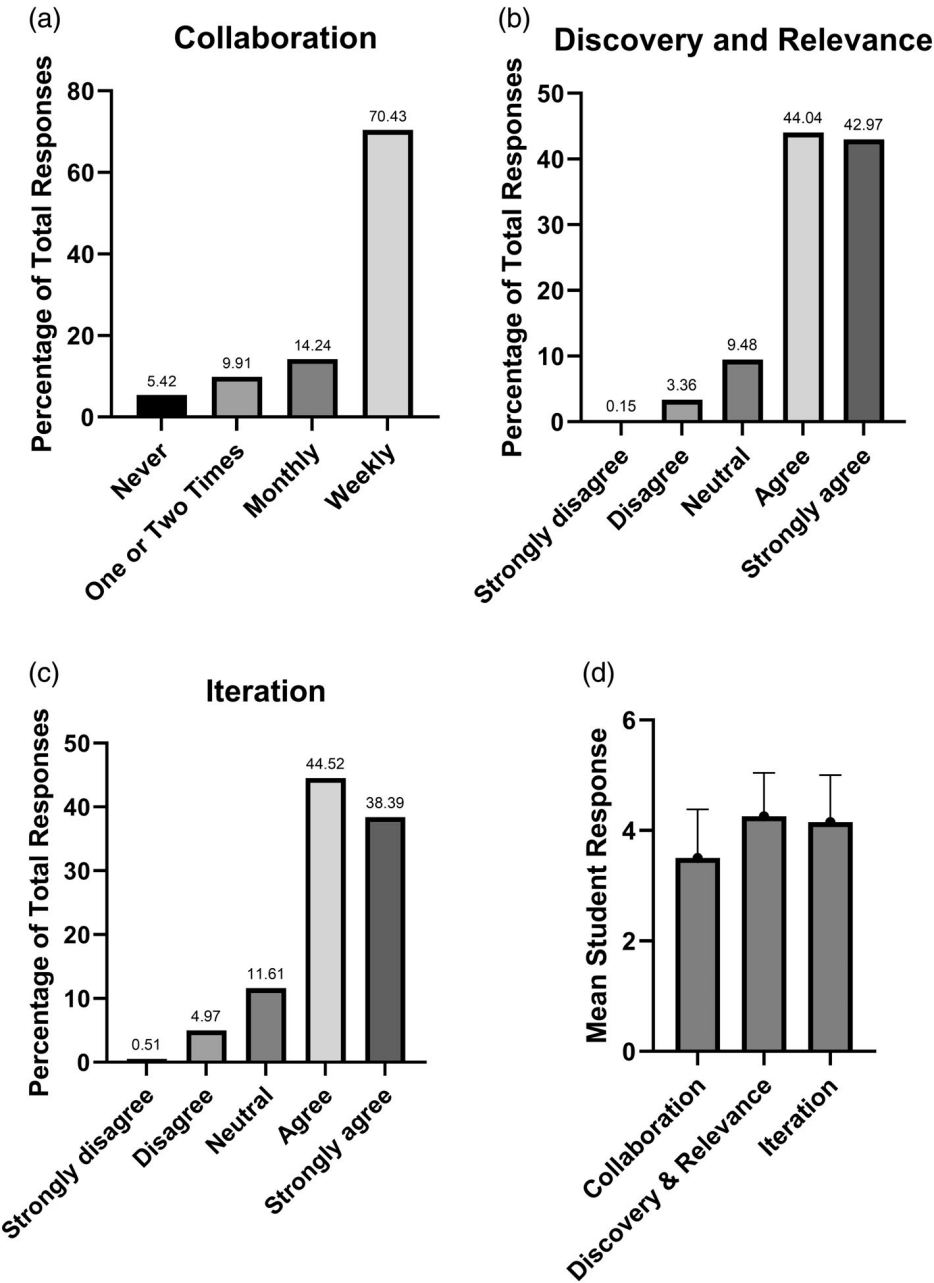
Results of LCAS reveal positive student experience in scientific collaboration (a), discovery and relevance (b), and research iteration (c) throughout their SynBio CURE lab. Numbers above bars are the total percentage of responses in each Likert rating category. (d) Mean (±SD) of the collective data plotted by converting Likert scale to numerical scale where strongly agree = 5, agree = 4, neither agree nor disagree = 3, disagree = 2, and strongly disagree = 1 for all survey questions, except for LCAS‐collaboration. LCAS‐collaboration questions had a four‐point scale with 1 = never; 2 = once or twice; 3 = monthly; 4 = weekly. Hundred and thirty‐two students from five institutions completed this survey

When questioned how often they were encouraged to engage in particular collaborative aspects of the course, such as discuss elements of one's research with peers and instructors and contribute ideas and suggestions during class discussions, 70% responded that they did so weekly (Figure 6a). The overall mean for collaboration was 3.5 out of 4 (+/− 0.88), indicating an average of collaborative activities between monthly and weekly (Table 2; Figure 6d). While the 70% weekly response was a composite answer, we saw an outlier in the individual collaboration questions. One specific question probes the idea of providing constructive criticism to classmates and how often students challenged each other's interpretations. Here, only 40% of students said they were encouraged to critique other's work on a weekly basis. We found an even distribution, 20% each, between students who indicated they were encouraged to critique work monthly, one or two times over the semester, or never encouraged to do this. Anecdotally, students associate critique and constructive criticism as a negative, and subsequently avoid the topic. Additionally, it is our experience that faculty tend to shy away from requiring students to critique each in order to reduce conflict. This aspect is one where we can target improvement in the future.

The second section of the LCAS focuses on student engagement in particular activities related to discovery and relevance. For example, students are asked to rate their level of agreement with statements, such as “In this course, I am expected to… generate novel results, …formulate my own research questions, and …conduct an investigation to find something previously unknown.” The mean student response for the discovery and relevance questions was 4.26 out of 5 (+/− 0.78) (Table 2; Figure 6d), indicating an average response that was somewhat above “Agree.” In fact, most students (87%) agreed or strongly agreed with statements indicating their lab work could lead to discovery of something new, development of new arguments, or generation of knowledge of interest to the scientific community (Figure 6b).

The SynBio lab was designed with a limited number of basic lab techniques (Figure 1; Table 1). The intent was to give students time for iteration of techniques. Iteration is an important part of scientific inquiry because new knowledge builds from existing knowledge.^22^ Through iteration, students are able to reflect on what went wrong in an experiment and make corrections to prevent future mistakes. Additionally, the iteration section of the LCAS examines student experiences comparing data with other students and making revisions in research processes based upon feedback, both of which are actively incorporated into the structure of the SynBio CURE. Mean responses for iteration were 4.15 out of 5 (+/− 0.85), indicating an average response that was slightly higher than “Agree” (Table 2; Figure 6d). In agreement with this, 83% of the students surveyed agreed or strongly agreed they had time or direction to repeat aspects of their work, such as making revisions, changing methods, and analyzing additional data (Figure 6c).

### 3.2 Evidence of student persistence in science

A key tenant of a CURE is engaging students in authentic research as a means to build confidence and increase the likelihood of being retained in a STEM field.^5^, ^23^ To examine this, we used the Persistence in the Sciences (PITS) assessment survey.^18^ The PITS survey captures changes that promote undergraduate persistence in science, technology, engineering, and mathematics (STEM) fields.^18^ The PITS survey incorporates a six‐factor structure consisting of project ownership (emotion and content), self‐efficacy, science identity, scientific community values, and networking. While the SynBio CURE has been conducted at multiple institutions, due to IRB restrictions and COVID‐19 related campus lockdowns, we could only assess classes at the University of New Hampshire‐Manchester (UNH) and Ouachita Baptist University (OBU). Participants included 18 male and 28 female students. Most students were white with two students identifying as Hispanic/Latino, one as Black or African American and one indicating other. Participants included 24 seniors, 17 juniors, and 5 sophomores.

**FIGURE 7:**
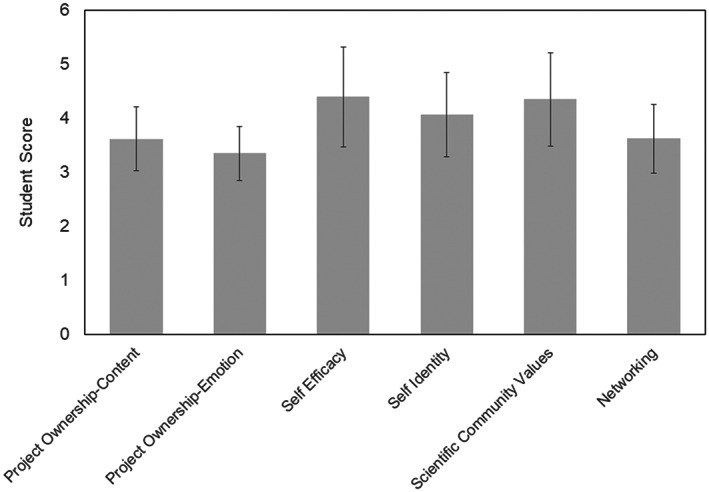
PITS survey data reveals student gains in all six categories indicating that students are engaged in the scientific practices that set CUREs apart from traditional labs. The PITS survey rating scales are from one (strongly disagree) to five (strongly agree) for all measures except for scientific community values, in which ratings are one (not like me at all) to six (very much like me). Total *n* = 46; UNH students *n* = 16; OBU students *n* = 30. Data are represented as mean ±SD

In all six categories, students in the SynBio CURE demonstrated similar scores to CURE students surveyed in the 2017 Hanauer iREC study thus outperforming students enrolled in traditional labs (Figure 7).^24^ For example, students agreed with statements indicating high levels of self‐identity, thinking of oneself as a scientist, and self‐efficacy, confidence in functioning as a scientist.^25^, ^26^ Project ownership indicating the degree of a student's personal engagement with the content as an indicator of an authentic research experience was another area in which students scored highly. Additionally, Hanauer et al.^24^ indicated the percentage of students that score below or above a threshold in any one section may be indicative of the overall perception of the lab. The lowest mean student response was for project ownership (3.7 content and 3.36 emotion). However, 50% of the surveyed students rated their engagement with as a 4 (agree) or 5 (strongly agree) for these sections of project ownership. The dichotomy of student responses in this section indicated they were either highly emotionally invested or they demonstrated little investment in the CURE experience.

## 4 SAFETY AND ETHICAL ISSUES

Students should wear gloves and safety glasses while performing these experiments. The suggested bacterial chassis utilized in this lab is JM109, a BSL‐1 strain. Biohazard bag disposal of BSL‐1 bacteria and media is required. Otherwise, all other reagents are non‐toxic and may be disposed of in the sink. For gel electrophoresis, it is recommended to use safe DNA stains, such as GelRed or SybrSafe. Both DNA bands in gels and fluorescent protein‐expressing bacteria are recommended to be visualized under blue light. If UV light is utilized to visualize gels or fluorescent colonies, appropriate UV‐blocking shields should be worn by all personnel. There are no obvious ethical issues associated with this research.

## 5 DISCUSSION

Traditional models of incorporating undergraduate students into research labs are limited, due to lack of availability, mentor time, space, and mentorship resources. CUREs provide an excellent opportunity for undergraduate students to engage in authentic research. The CURE model enables more students to take ownership over an independent research project and learn important critical thinking skills. The SynBio CURE allows students to work toward a consistent goal utilizing standardized molecular cloning techniques and promotes problem‐solving while reveling in the excitement of designing and engineering a novel bacterial device.

Because the SynBio CURE utilizes bacteria and molecular biology techniques, which are very amenable to easy start and stop points and long‐term storage, the CURE can be employed in a variety of timelines and implementation strategies. While it is outlined here as part of a whole semester lab course, it can and has been utilized to introduce these experiments to students in shorter modules to suit different course structures and curricula.

It is evident through the LCAS that the SynBio CURE supports student engagement in most CURE aspects on a weekly basis. The exception is with students constructively critiquing each other which is an important skill for scientists to develop. This will be something we will focus on as an area to improve in the future. Students also either agreed or strongly agreed that they were able to engage in CURE‐related activities during the semester and, importantly, that they were able to engage in multiple iterations of the activities. We can conclude that the SynBio CURE is meeting the important aspects that distinguish CUREs from traditional lab courses.

The PITS survey revealed that students in the SynBio CUREs exhibit gains in all six categories of project ownership, self‐efficacy, science identity, scientific community values, and networking. Although we did not compare our results to those from students enrolled in our own traditional lab courses, our results are comparable to those from Hanauer's iREC study which concluded CURE students outperform traditional lab students in all six categories.^24^ Thus, the SynBio CURE contains the elements that separate CUREs from traditional labs and likely engage students in scientific practices to a larger extent than traditional labs.

The SynBio CURE as described was done with undergraduates at one small private PUI and two regional state institutions. However, as result of the Arkansas‐CURE workshops the lab has been implemented and modified to fit a wide range of institutional needs. While each institution uses similar techniques, the research projects vary. It is our goal to provide students and faculty with a framework for a synthetic biology CURE rather than create a uniform prescribed research question. In this way, the SynBio CURE can be modified to fit a range of needs, curricular goals, and research interests.

## CONFLICT OF INTEREST

The authors have no conflict of interest, financial or otherwise.

## Supporting information


**Supplementary Figure S1** Location of institutions that attended the AR‐CURE workshop between 2017 and 2020. Blue stars represent 2017 participants, red stars 2018 participants, and green stars 2019 participants. Gold stars represent 2020 virtual participants.
**Supplementary Table 1. Common themes in implementation of Synthetic Biology CURE between different institutions.** Implementation of the SynBio CURE is very similar among the three institutions cited in this manuscript with slight variations highlighted here. The activities and assessments listed in this table, including example student work, are provided through documents in the Supplementary Materials.
