# Supplementary material for: From genetics to biotechnology: Synthetic biology as a flexible course‐embedded research experience
Source: Biochem Mol Biol Educ. 2022 Sep 2;50(6):580–91. doi: 10.1002/bmb.21662 (PMC9826443; doi:10.1002/bmb.21662)
Supplement: Supplementary file 1 — Supplementary Figure S1 Location of institutions that attended the AR‐CURE workshop between 2017 and 2020. Blue stars represent 2017 participants, red stars 2018 participants, and green stars 2019 participants. Gold stars represent 2020 virtual participants. Supplementary Table 1. Common themes in implementation of Synthetic Biology CURE between different institutions. Implementation of the SynBio CURE is very similar among the three institutions cited in this manuscript with slight variations highlighted here. The activities and assessments listed in this table, including example student work, are provided through documents in the Supplementary Materials. [file BMB-50-580-s001.docx]

Supplementary Material

- Supplementary Figure 1
- Supplementary Table 1

**Assignments, Powerpoints, and Templates for Student Work:**

1. Synthetic Biology Project Primer – Restriction Enzyme Activity: Introduction to iGEM projects as well as the restrictions enzymes and plasmids used for cloning in this lab.
2. How to Use the iGEM Registry (table/spreadsheet) – Used as a tool to help students navigate the Registry of Standard Biological Parts, iGEM.
3. BioBrick Assembly powerpoint and activity – Teaching tool for how 3A assembly works as well as slides to print for students to cut and paste (using scissors and tape) to model the restriction digest and ligation that happens during the 3A assembly.
4. Project Proposal Template and Grading Rubric (powerpoint)
5. Final Presentation Template (powerpoint)
6. Device Analysis Paper Assignment and Rubric

**Examples of Student Work:**

1. Project Proposal Presentation
2. Project Update Presentation
3. Final Project Presentation

**Supplementary Figure 1.** Location of institutions that attended the AR-CURE workshop between 2017-2020. Blue stars represent 2017 participants, red stars 2018 participants, and green stars 2019 participants. Gold stars represent 2020 virtual participants.


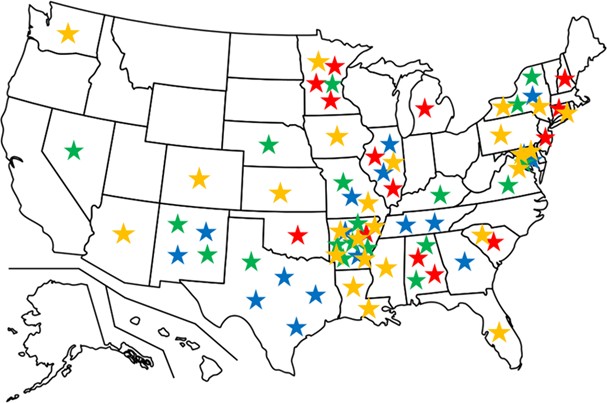


**Supplementary Table 1. Common themes in implementation of Synthetic Biology CURE between different institutions.** Implementation of the SynBio CURE is very similar among the three institutions cited in this manuscript with slight variations highlighted here. The activities and assessments listed in this table, including example student work, are provided through documents in the Supplementary Materials.

| ***Activity/ Assessment:*** | iGEM  website survey | Restriction enzyme assignment/  worksheet | BioBrick assembly  / cut paste  activity | Research outline/ proposal | Lab Notebooks | | Group lab  meetings | Lab Presentations (power point) | | | poster presentation | research paper |
| --- | --- | --- | --- | --- | --- | --- | --- | --- | --- | --- | --- | --- |
| **Institution (Instructor)** |  |  |  |  | Online notebook | Physical Notebook |  | Intro | project update | final data  analysis |  |  |
| OBU (Reyna) |  |  |  | *outline* |  |  |  |  |  |  |  |  |
| UNH (Johnson) |  |  |  | *proposal* | *Benchling* |  |  |  |  |  |  |  |
| UMemphis (Sabel/Cole) |  |  |  | *outline* |  |  |  |  | weekly lab reports |  |  |  |

Team Members: Synthetic Biology Project Primer: Lab 1

**Complete in Teams of 2-4**

1. Find one example of something that was done at a previous competition. Look how their wiki is organized. Briefly describe the goals of their project. Briefly describe one functional device that was created as a result of this project. <http://igem.org/Previous_Competitions>

(Write some summary notes here; you will report out as a team to the rest of the lab.)

1. Go to the registry of standardized parts. <http://parts.igem.org/Main_Page>. Take a few minutes and look around this website. This will be one of the main websites we will use in class.
2. List the 4 restriction endonucleases used in basic bio bricks (BBI)/iGEM. What are the recognition sequences for each? Indicate the sticky ends found that would be created for each. This is sometimes referred to as the bio- brick standard.
3. What is a linear plasmid backbone? What are the four different backbones (antibiotics) that are commonly used?
4. I only have access to the 2018 distribution (kit) plates. For lab, you have to identify parts that can be obtained from these plates. You will find information about promoters, reporter proteins and ribosome binding sequences that potentially could be used in class. Use the “iGEM Website Survey” file (MyCourses download) to complete a search and find to help you learn how to use the Registry of Standardized Parts.

Hint: one of the easiest ways to do this is to look at the collection website:

Name / part number/ link to website / Size in base pairs / Location on 2018 kit plate / Brief description of previous success / description of part

**You will need to download the** iGEM Website Survey **Spreadsheet excel file from MyCourses and complete the columns.**

| **Typ e** | **Name** | **Part Number** | **size (bp)** | **Location on 2018 Kit plate** | **Descript ion of Part.** | **How many others have used it?**  **(success)** | **Link to part informatio n** |
| --- | --- | --- | --- | --- | --- | --- | --- |

1. Compare and combine your results with the results of another lab team. *Be ready to discuss your findings.*
2. E-mail me this list (along with the names of the members of your team).
3. What type of device might you like to make? (add parts to your list)
4. Make a list of questions you still have.

**Student Activity: Using the information in the iGEM Registry of Standard Biological Parts (**[**http://parts.igem.org/Main_Page),**](http://parts.igem.org/Main_Page)) **search for and fill in the following information that will help you learn how to use the registry to find parts for your own device design.**

**Find one part in the 2021 iGEM distribution**

| **Part ID** | **Name** | **size** | **well**  **location** | **Year-**  **Plate** | **plasmid**  **backbone** | **type** | **description** | **Link** |
| --- | --- | --- | --- | --- | --- | --- | --- | --- |
| BBa_K808000 | AraC-Pbad | 1209 | 7E | 2018-  plate 2 | pSB1C3 | promoter | Arabinose inducible promoter with regulator. | http://parts.igem.org/Part:B Ba_K808000 |
| BBa_J04450 |  |  |  |  |  |  |  |  |
|  |  |  |  |  |  | RBS | Ribosome Binding Sequence |  |
|  | amilCP |  |  |  |  |  |  |  |
| BBa_K515005 |  |  |  |  |  |  |  |  |

Links to the parts and plasmids is optional but helps students quickly access information later.

| **Part ID** | **Name** | **size** | **well**  **location** | **Year-**  **Plate** | **plasmid**  **backbone** | **type** | **description** | **Link** |
| --- | --- | --- | --- | --- | --- | --- | --- | --- |
|  |  |  |  |  |  |  |  |  |

| **Plasmid** | **Antibiotic Resistance** | **size** | **iGem link (Optional)** |
| --- | --- | --- | --- |
| pSB1C3 | chloramphenicol | 2070 |  |
| pSB1T3 |  |  |  |
| pSB1K3 |  |  |  |
| pSB1A3 |  |  |  |

BioBrick Assembly Lab Presentation

Basic Bio-Brick Assembly (BBA)

- 1. What is a Restriction endonuclease (restriction enzyme)?
  2. What are the four restriction (cut) sites used in BBA?
  3. What are their specific recognition sequences and where does each enzyme cut?
  4. How are they oriented on a Bio-Brick?

What are the four “cut” sites for Basic Bio-Brick Assembly (BBa)? *[look them up and write them down]*

Where (how) does the restriction enzyme cut the DNA?

BioBrick Restriction Enzymes

- EcoRI (E)
- PstI (P)
- SpeI (S)
- XbaI (X)

Basic Bio-Brick Assembly (BBA)

and Orientation of Cut Sites

BioBricks

**BioBrick Part**

E

X

S P

origin

antibiotic resistance

BioBrick plasmid backbone + 1 part

**BBa_B0015**

BioBrick plasmid backbone

[(http://partsregistry.org/Plasmids)](http://partsregistry.org/Plasmids)

BioBricks

E

X

S

P

antibiotic resistance

origin

Part A

E

X

S

P

antibiotic resistance

origin

Part B

**Traditional Genetic Engineering vs.**

**BioBrick Assembly**

- - Construction of recombinant DNA tailored to specific projects
  - Natural occurrence of restriction sites dictates assembly
  - Assembly is often complex

BioBricks

E

X

S

P

antibiotic resistance

origin

Part A

E

X

S

P

antibiotic resistance

origin

Part B

Exercise: Using the following slide handouts, and scissors (restriction enzymes) and tape (ligase), perform biobrick assembly in order to clone part A before part B.

- Use restriction endonucleases (scissors) to cut the sequence for example, with **PstI** as follows:

C T G C A G G A C G T C

- GOAL: Ligate BioBrick part A and BioBrick part B together (in that order: A must come before B!) within a single backbone plasmid.

**Plasmid Prefix**

### EcoRI XbaI SpeI PstI

**Plasmid Sufix**

GAATTC—TCTAGA

Part A

ACTAGT--CTGCAG

CTTAAG—AGATCT TGATCA--GACGTC


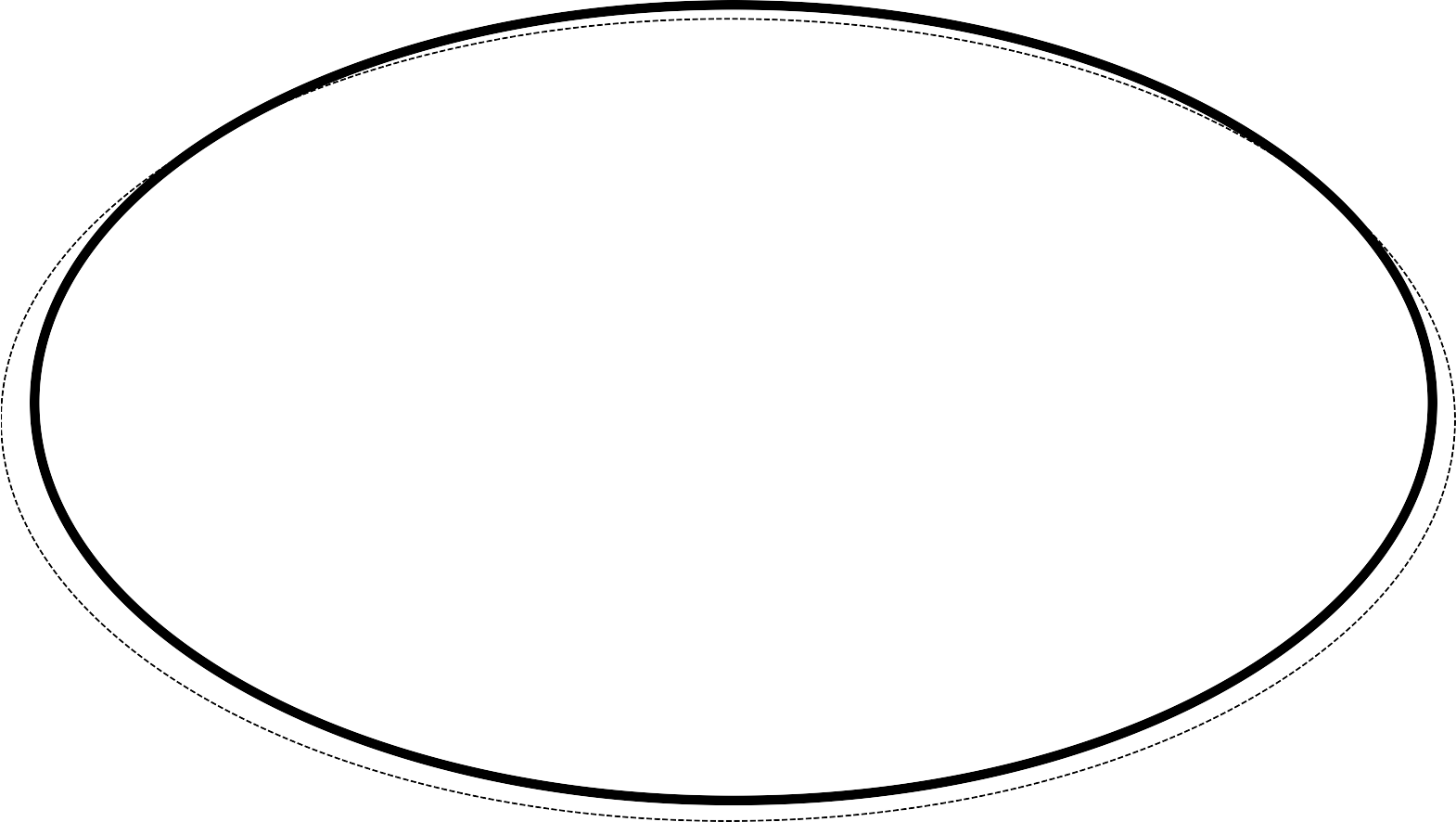


**PLASMID_**Chloramphenicol

Cut on dotted line

### EcoRI XbaI SpeI PstI


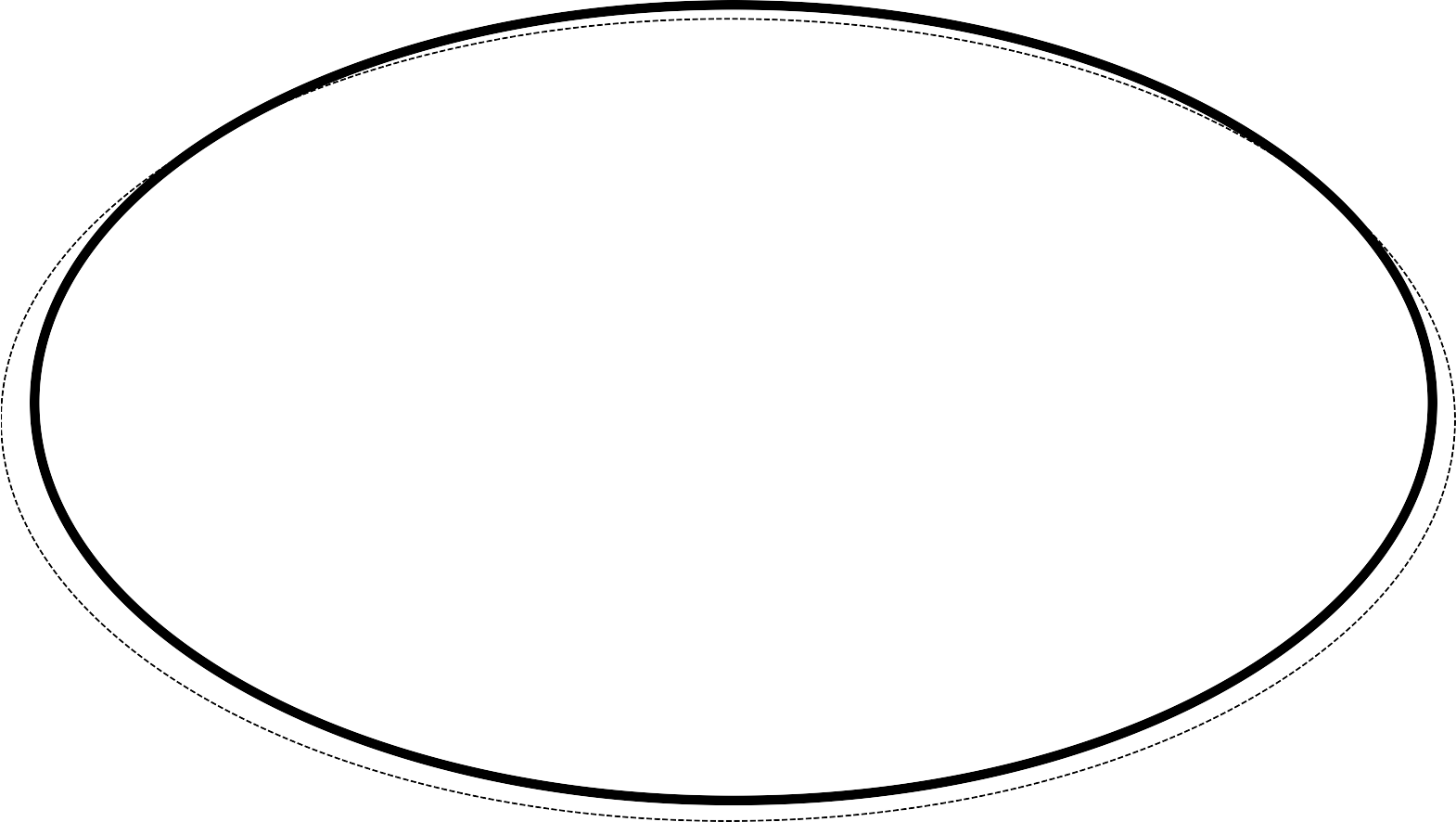


|  | | |  |
| --- | --- | --- | --- |
|  | GAATTC—TCTAGA **Part B** |  |  |
|  | CTTAAG—AGATCT | TGATCA--GACGTC |  |

**PLASMID-**Chloramphenicol

Cut on dotted line

**CONGRATULATIONS: You have connected two BioBricks!**

- - Here’s the good part:
    - Does it have only one site for EcoRI, XbaI, SpeI, and PstI?
    - What happened to the XbaI and SpeI sites at the junction between the two parts?

BioBricks

E

X

S

P

antibiotic resistance

origin

Part A

E

X

S

P

antibiotic resistance

origin

Part B

BioBricks

S

P

E

X

S

P

antibiotic resistance

origin

Part A

X

Part B

BioBricks

E

X

SX

S

**P**

antibiotic resistance

origin

Part B

Part A

BioBricks

SX

E

X

S

P

antibiotic resistance

origin

Part B

Part A

BioBricks

E

X

S

P

antibiotic resistance

origin

Part A

E

X

S

P

antibiotic resistance

origin

Part B

E

X

SX

S

P

antibiotic resistance

origin

Part B

Part A

Observations?

- Biobrick ends
  -
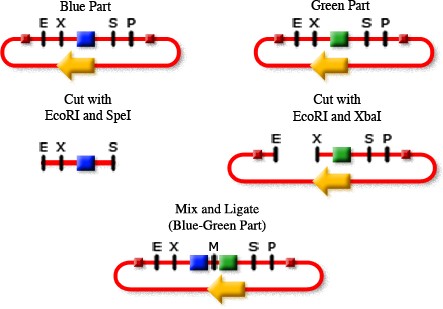
Prefix: E, X
  - Suffix: S, P
- X,S compatibility
- Insert before or after
- biobricks lead to biobricks

3 (A) Antibiotic Assembly

What is the purpose of antibiotic resistance?

What are the 4 most common antibiotic resistance genes used in BBA/3A assembly?

•

•

•

•

Ampicillin

Kanamycin Chloramphenicol Tetracycline

Student Exercise:

- - GOAL: Using the following handouts and BBA methods, assemble two different biobrick parts (e.g., a promoter and an RBS) into a third plasmid backbone.
  - Achieved this? Try to assemble 3 parts (promoter-RBS- coding) into another backbone.

### EcoRI XbaI SpeI PstI

**Plasmid Prefix**

## GAATTC--TCTAGA--ACTAGT--CTGCAG CTTAAG--AGATCT--TGATCA--GACGTC

**Plasmid Sufix**


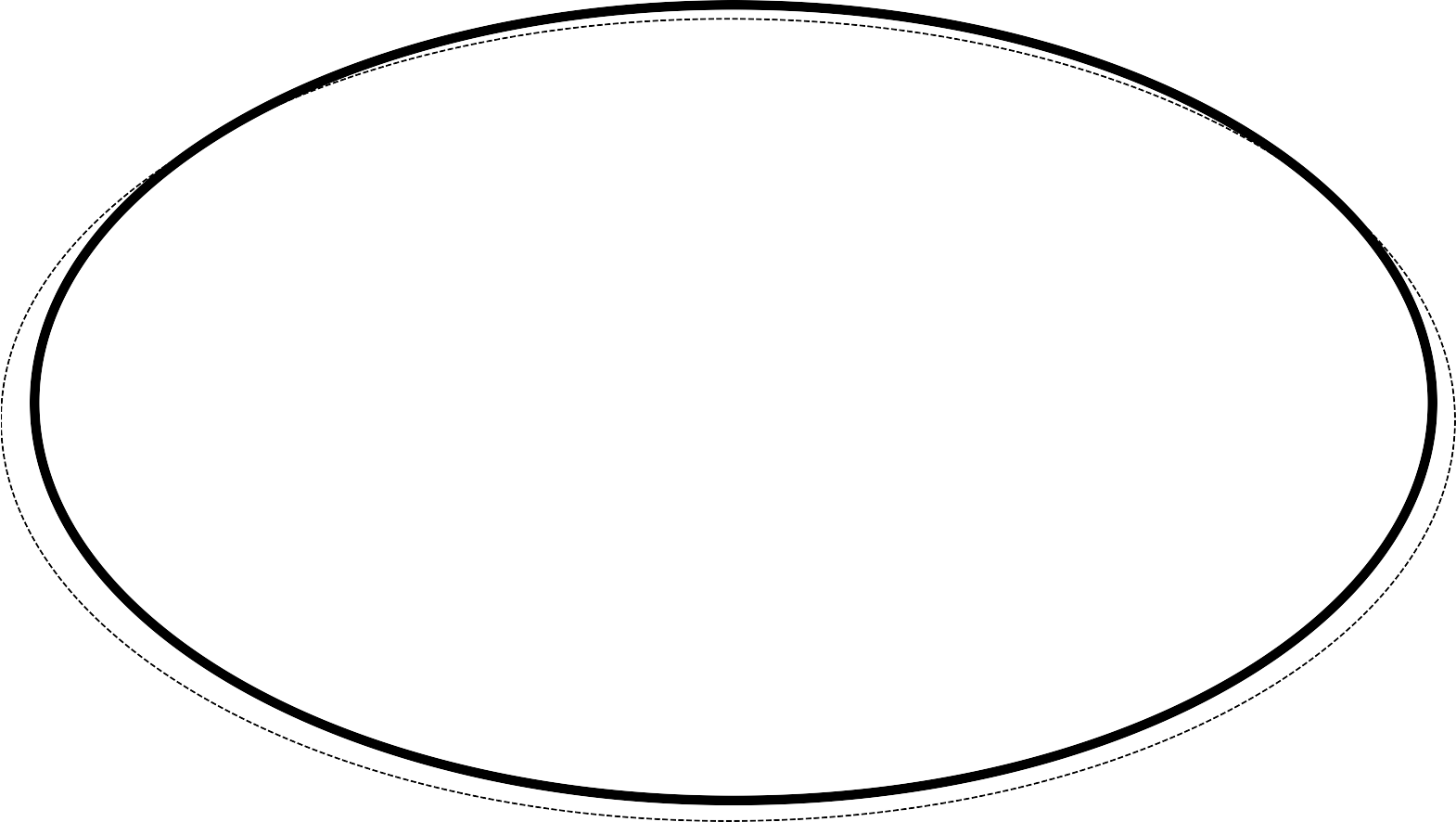


# Linear Backbone Kan

Cut on dotted line

### EcoRI XbaI SpeI PstI

**Plasmid Prefix**

## GAATTC--TCTAGA--ACTAGT--CTGCAG CTTAAG--AGATCT--TGATCA--GACGTC

**Plasmid Sufix**


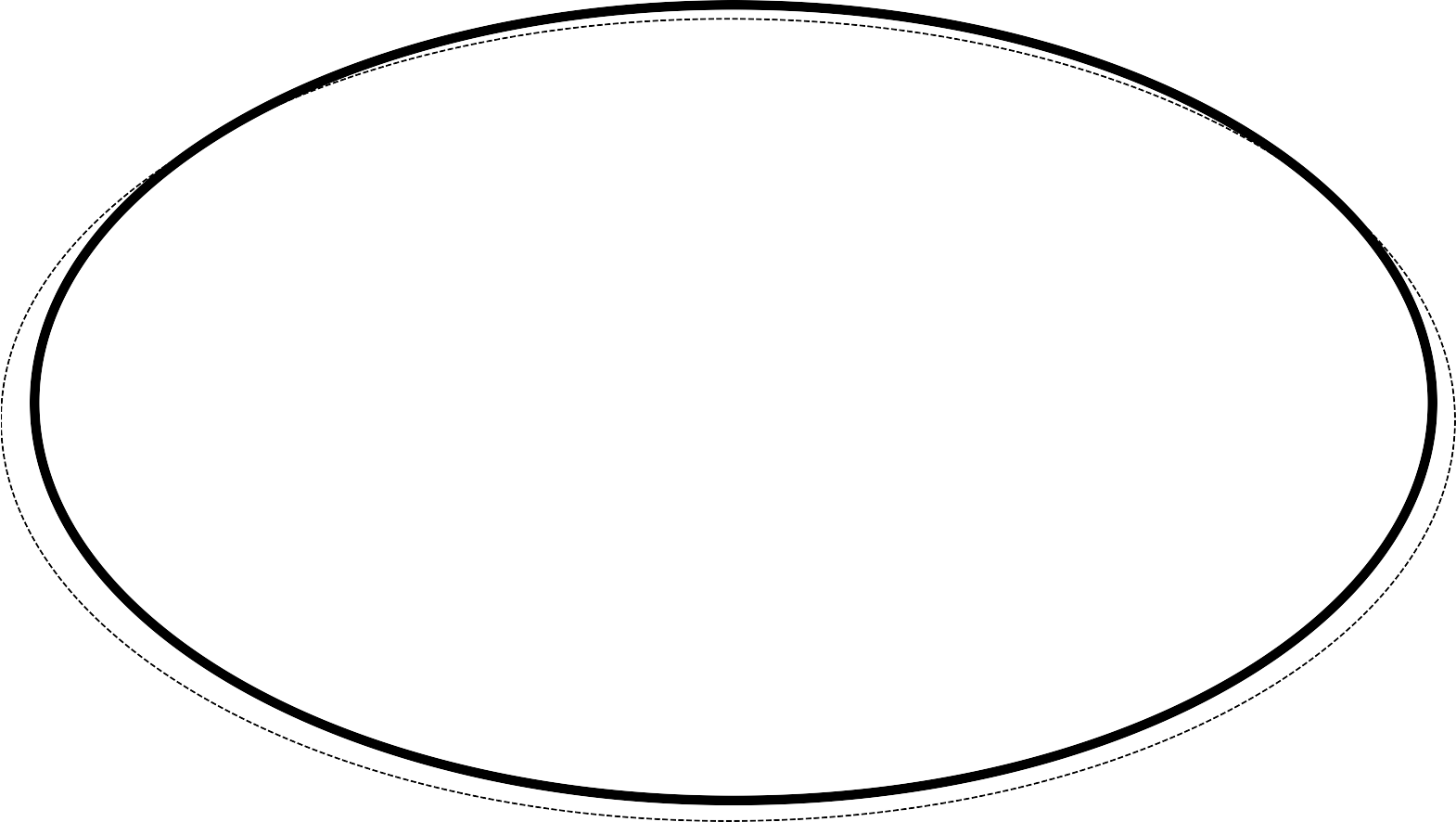


# Linear Backbone _ AMP

Cut on dotted line

### EcoRI XbaI SpeI PstI

**Plasmid Prefix**

## GAATTC--TCTAGA- -ACTAGT--CTGCAG CTTAAG--AGATCT- -TGATCA--GACGTC

**Plasmid Sufix**


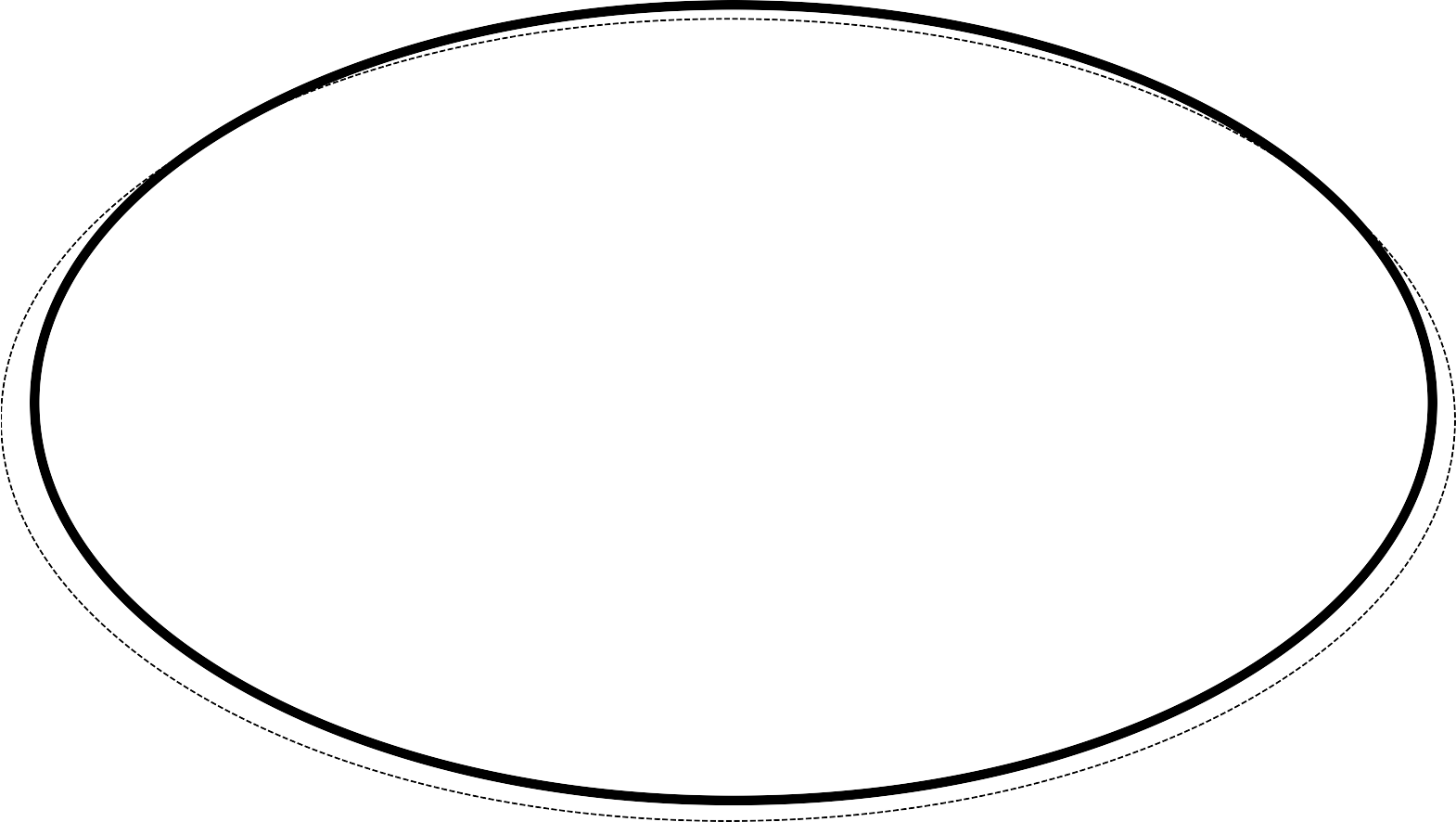


# Linear Backbone _ TET

Cut on dotted line

### EcoRI XbaI SpeI PstI

**Plasmid Prefix**

## GAATTC--TCTAGA--ACTAGT--CTGCAG CTTAAG--AGATCT--TGATCA--GACGTC

**Plasmid Sufix**


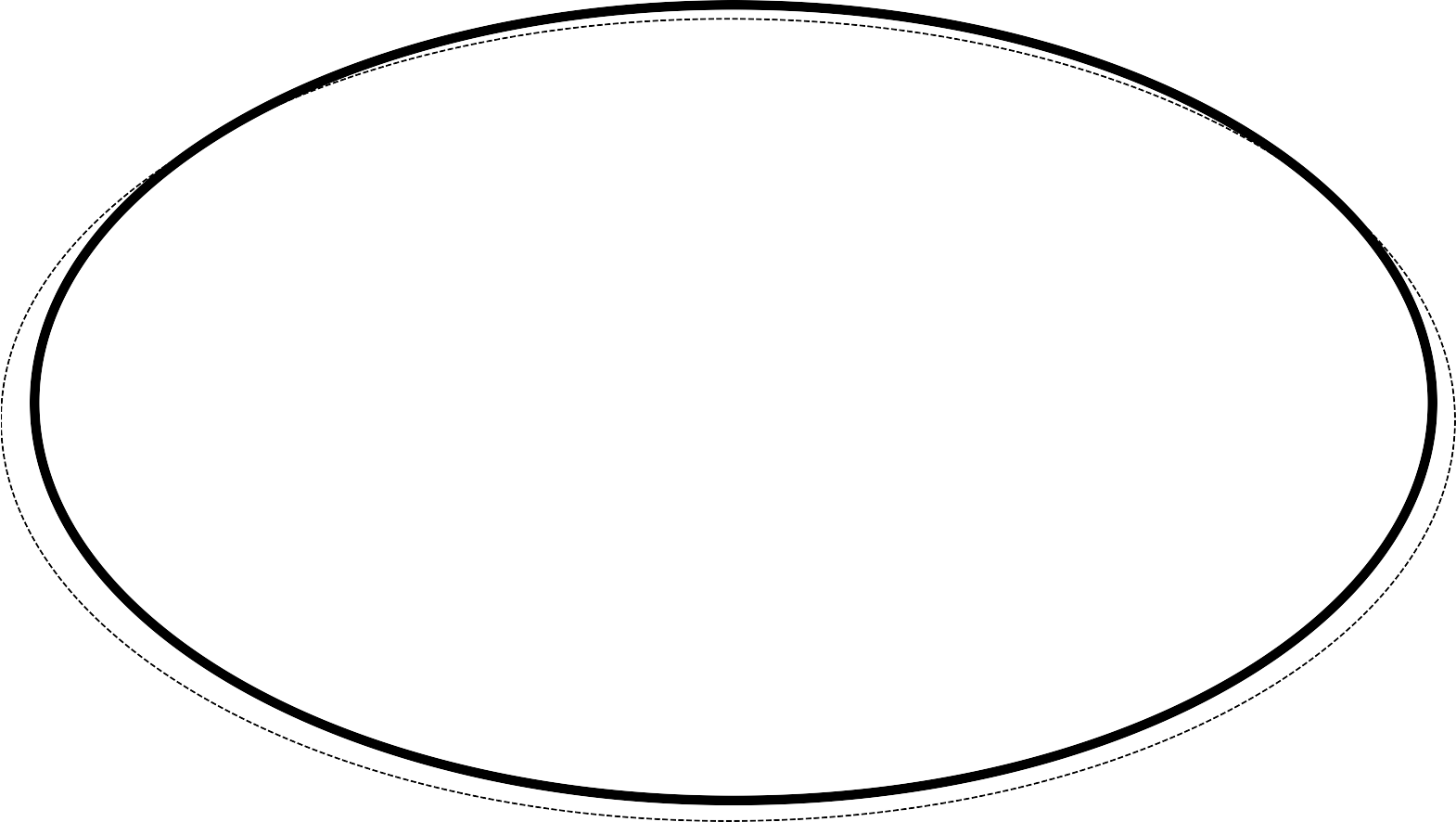


# Linear Backbone_

### Chloramphenicol

Cut on dotted line

EcoRI XbaI SpeI PstI

## GAATTC—TCTAGA ACTAGT--CTGCAG

**CTTAAG—AGATCT**

**promoter**

## TGATCA--GACGTC


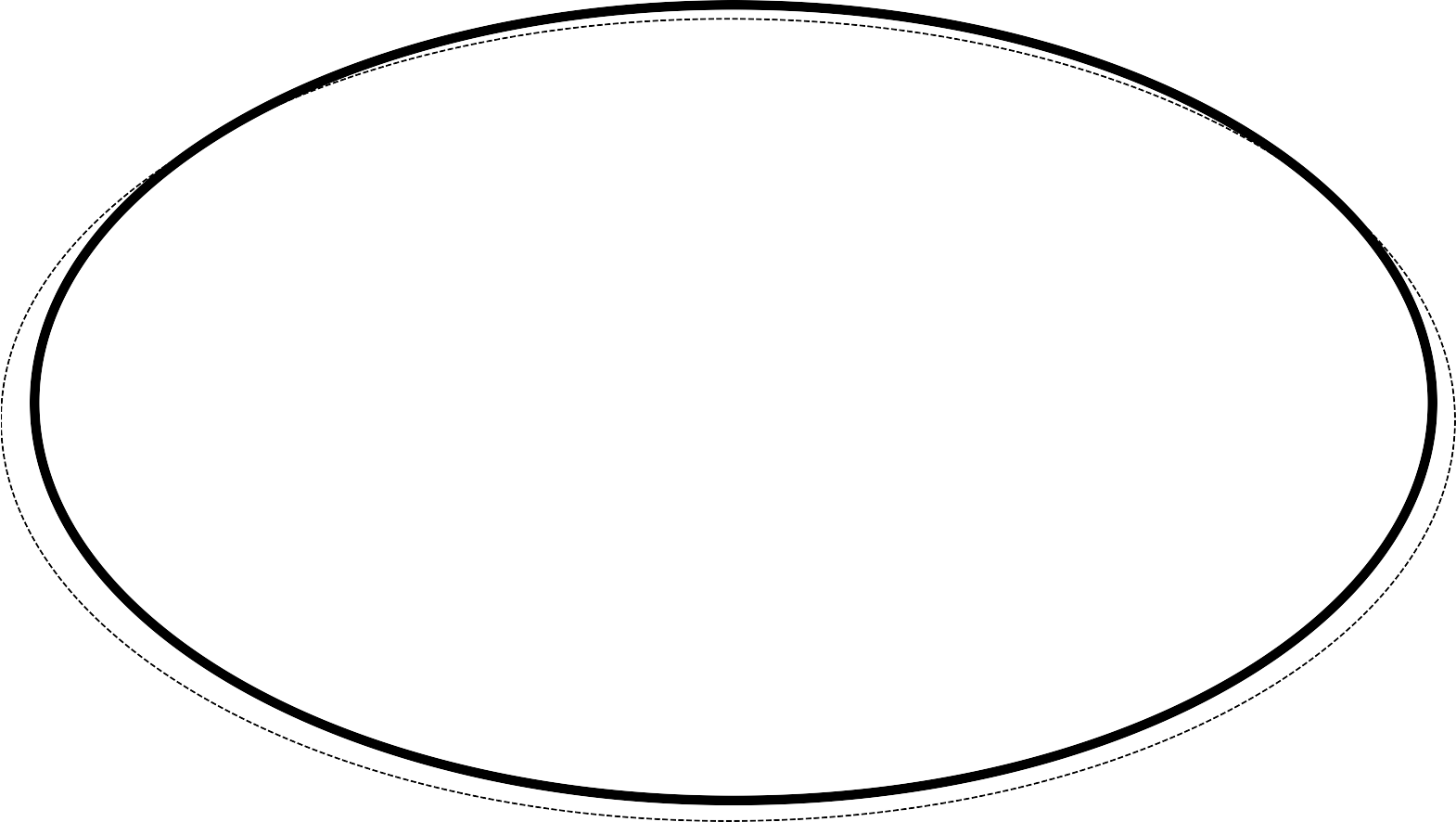

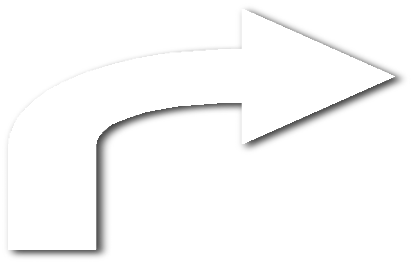


**PLASMID_**Chloramphenicol

Cut on dotted line

EcoRI XbaI SpeI PstI

## GAATTC—TCTAGA

RBS

## ACTAGT--CTGCAG


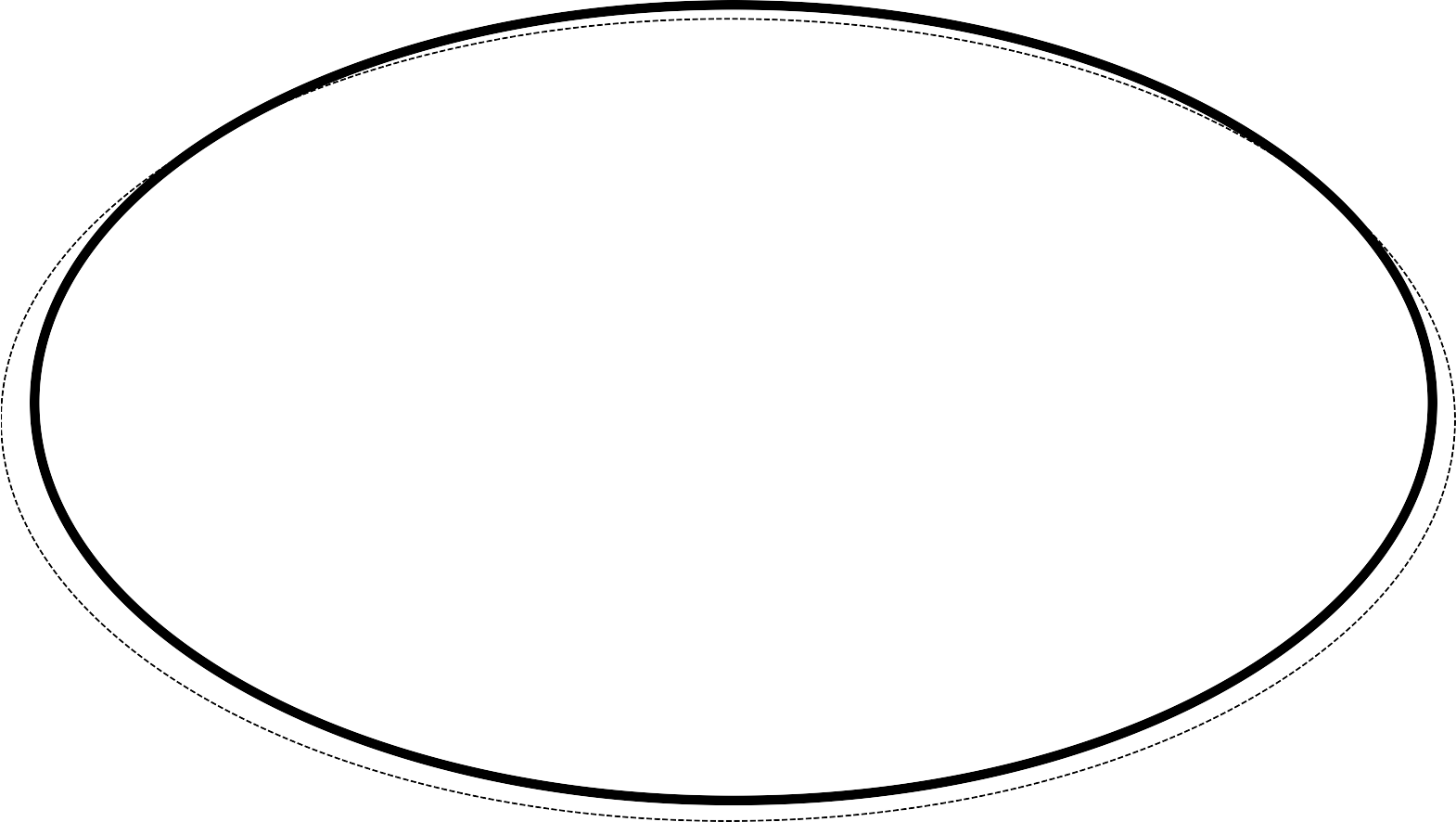


**CTTAAG—AGATCT TGATCA--GACGTC**

**PLASMID-**Chloramphenicol

Cut on dotted line

EcoRI XbaI SpeI PstI

## GAATTC—TCTAGA CTTAAG—AGATCT

GFP

**ACTAGT--CTGCAG TGATCA--GACGTC**


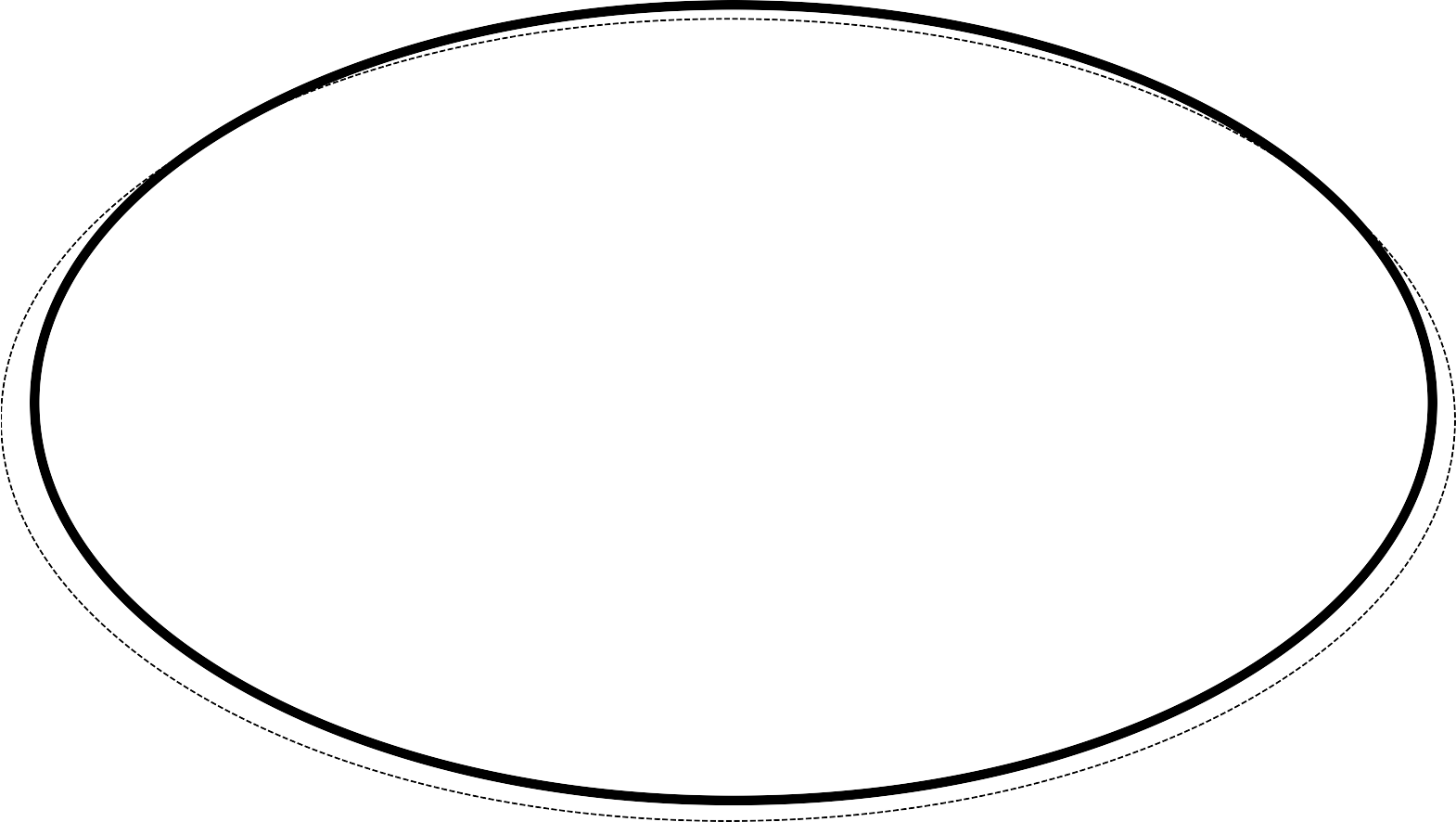


Cut on dotted line

# PLASMID-

Chloramphenicol

3 (A) Antibiotic Assembly Keys

- - Three Separate Plasmid Digestions
    1. Linear Backbone – Enzymes E&P
    2. Upstream Part (A) – Enzymes E&S
    3. Downstream Part (B) – Enzymes X&P


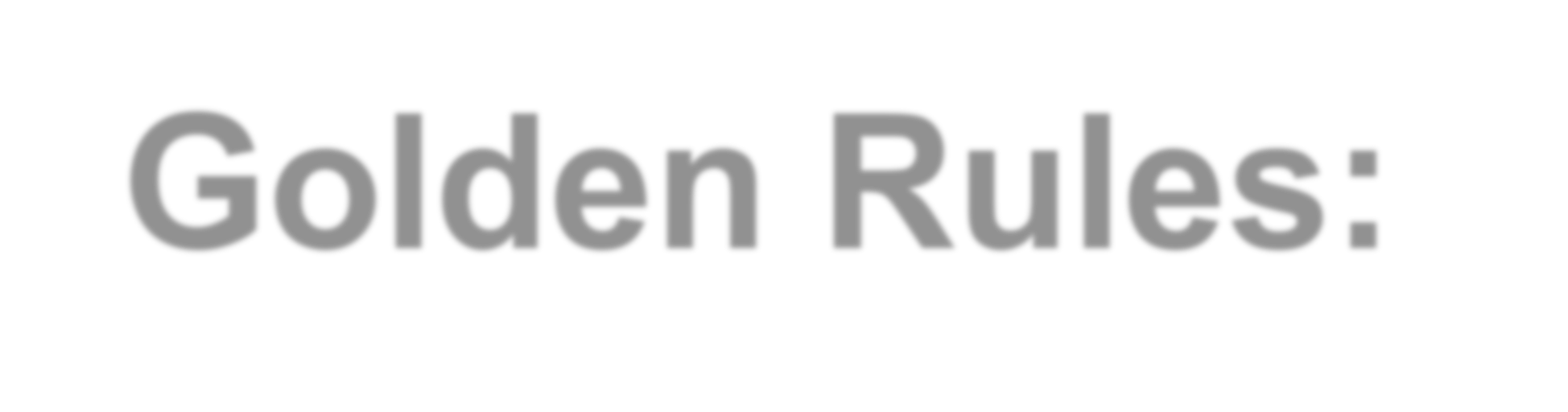

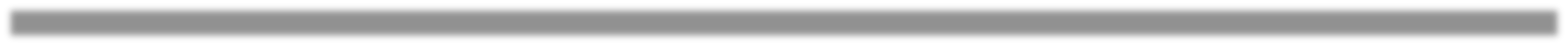


**Golden Rules:**

- - Linear Backbone must contain different antibiotic resistance than plasmid containing parts A and B. Why?
  - Must denature enzymes BEFORE you ligate.

3 A assembly


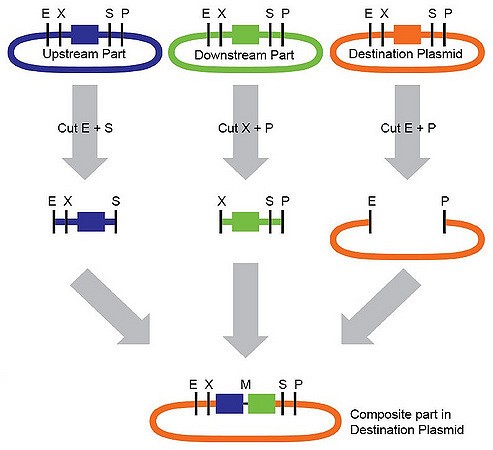


What should you ENSURE

about this

plasmid?

Linearized Destination Plasmid


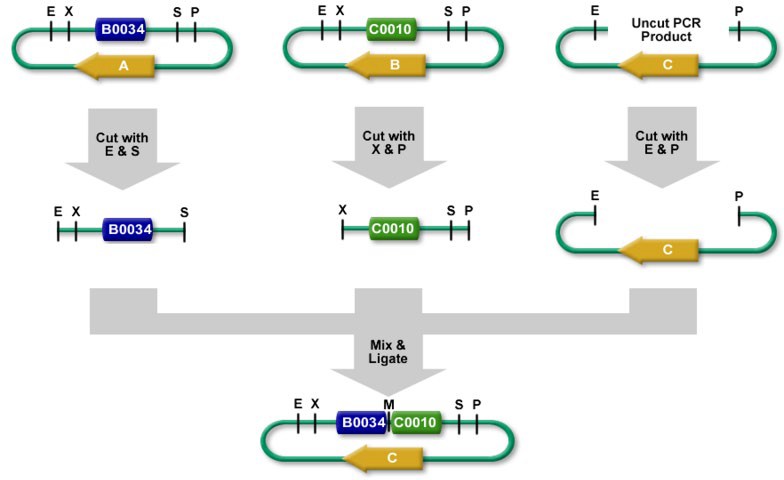


*antibiotic resistance gene*


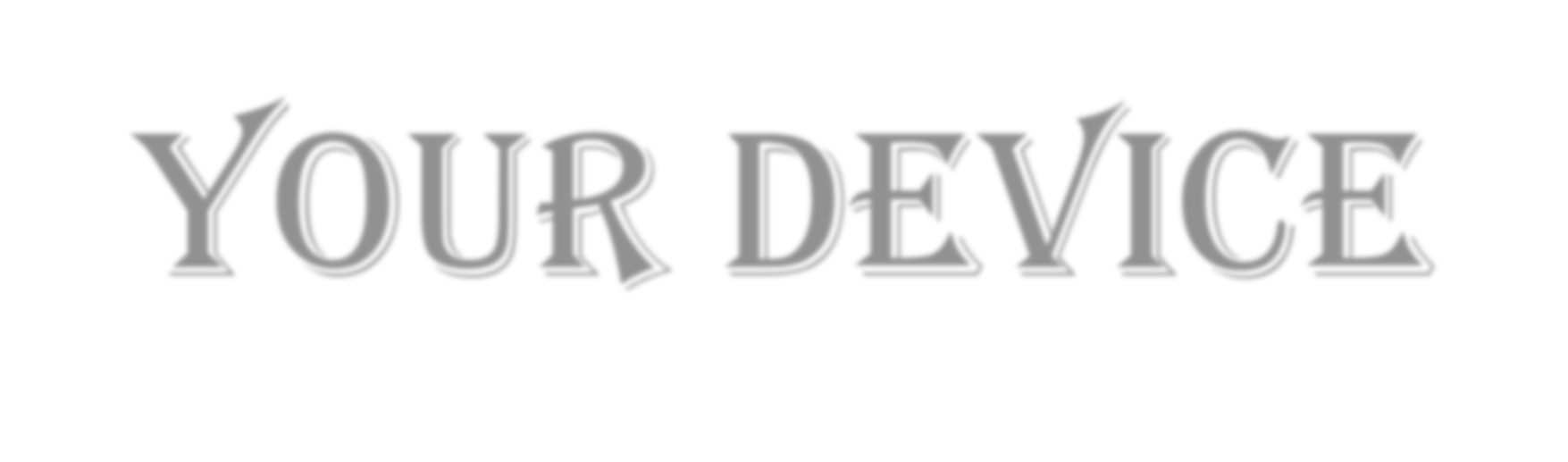
Now onto Your Device

- - What does your team want to build?
  - What parts do you need?
  - What is the work/experimental flow to build it and ensure you have the RIGHT parts put together in the right order?
  - How will you analyze your completed device?

Team Name’s

Project Proposal

TEAM LOGO

Presentation Template

##### Brief Summary of Your Project

First, describe the problem or challenge you are trying to solve.

Provide some background for the audience of why this is a problem and how you plan to solve it.

##### System Level Design: Truth Table and Chassis

- Chassis: *E. coli* (for all projects); JM109 strain (though you may have access to other strains, if needed)

Example:

| **Input** | **Output** |
| --- | --- |
| Absent | Off |
| Present | On |

##### Device Level Design: Abstraction

*Example:*

<5.5

>5.5

**pH DETECTOR**


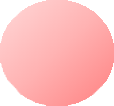


**Color GENERATOR**

##### Parts Level Design

*Example:*


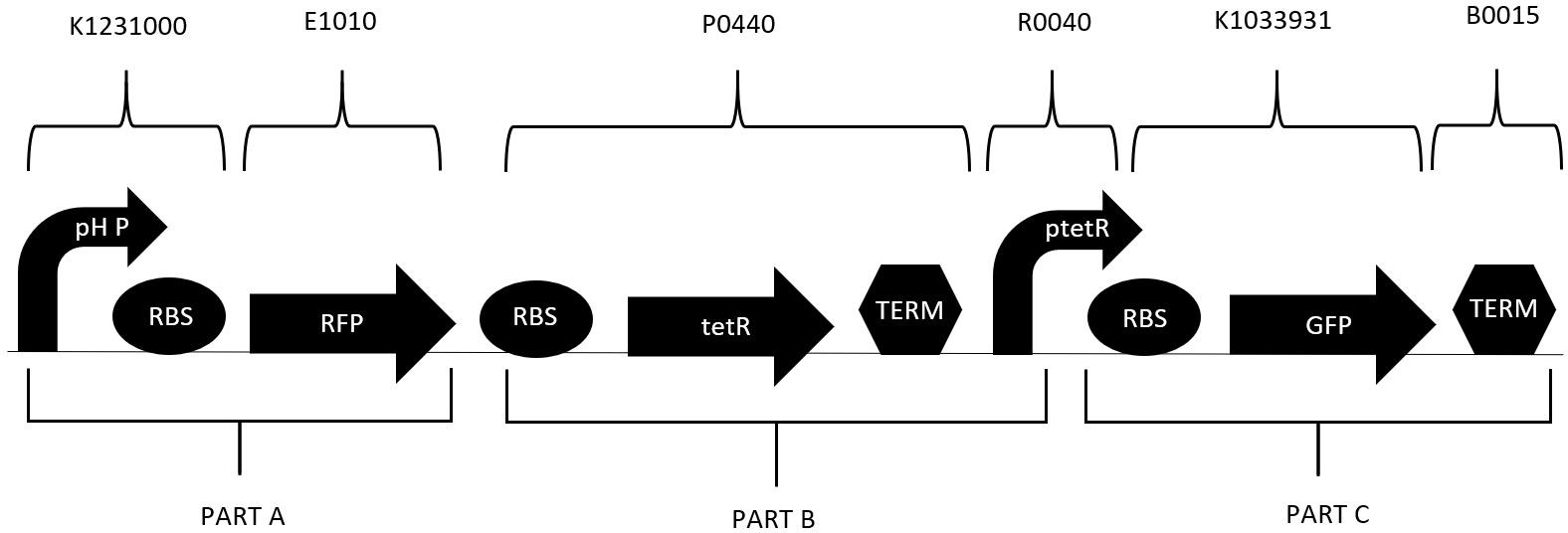


HIGH pH = GFP reporter

Tet inverter

LOW pH = RFP reporter


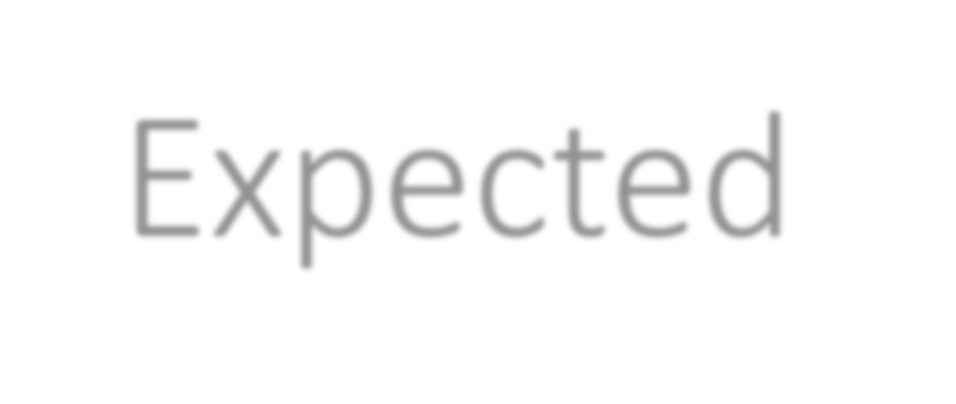

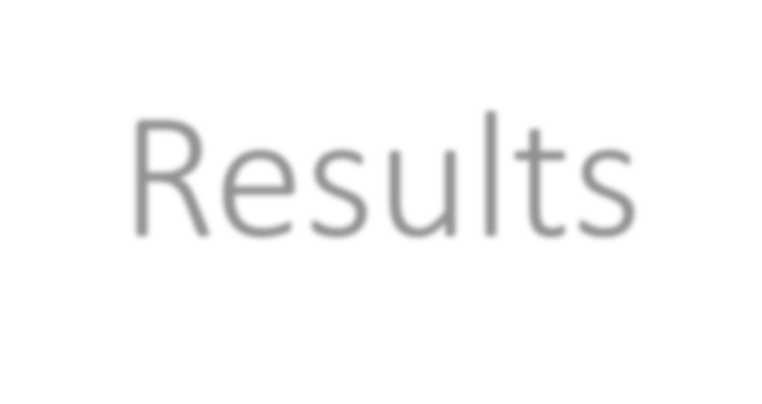


Expected Results

- What do you expect for results from your device as designed?
  - Describe/outline/diagram this

Analysis/Tests

*Describe:*

- How do you plan to test the efficacy of your device?
  - What specific experiments will be carried out to collect this data?
    - What supplies (reagents, concentration ranges, etc) will you need?
    - Will you test in liquid or solid media (or both)?
    - How will you analyze the output – qualitative and/or quantitative measures?

Problems?

- What are the most likely problems you anticipate encountering?
  - Be thorough in your considerations, here.
  - How might you overcome these problems?

Table of Parts – *what DNA sequences are you going to use to create your device?*

*-* Registry of Standard Biological Parts

| **Part ID** | **Name** | **size** | **well**  **location** | **2021 Year-**  **Plate** | **plasmid**  **backbone** | **type** | **description** |
| --- | --- | --- | --- | --- | --- | --- | --- |
|  |  |  |  |  |  |  |  |
|  |  |  |  |  |  |  |  |
|  |  |  |  |  |  |  |  |

*Are any (special) supplies needed to carry out the analysis phase? (e.g., isoamyl alcohol)*

##### Works Cited

**SynBio Lab Proposal Rubric**

**Team Proposal Score sheet Team Name**

| **Category** | Criteria | **Points** | **Student Evaluation** | **Instructor Evaluation** |
| --- | --- | --- | --- | --- |
| **Purpose** | Clearly stated. Presents background of  problem being addressed and solution. | 4 |  |  |
| **Design Description** | Thoroughly presents input(s) and output(s) with each design level. Displays insight  into project design. | 18 |  |  |
| **Expected results** | Multiple results are considered. | 6 |  |  |
| **Problems** | Most likely problems are thoroughly explained. | 3 |  |  |
| **Tests** | Well explained with details. Results of designed  tests will inform about efficacy of device. | 8 |  |  |
| **Mechanics** | Format: Proposal power point format is  followed. | 4 |  |  |
|  | Organization. Use of bullet points and images aid organization. | 3 |  |  |
|  | All grammar and spelling are correct. | 2 |  |  |
|  | Bibliography (APA format) | 2 |  |  |
|  | | 50 |  |  |

#### Final Presentation Template:


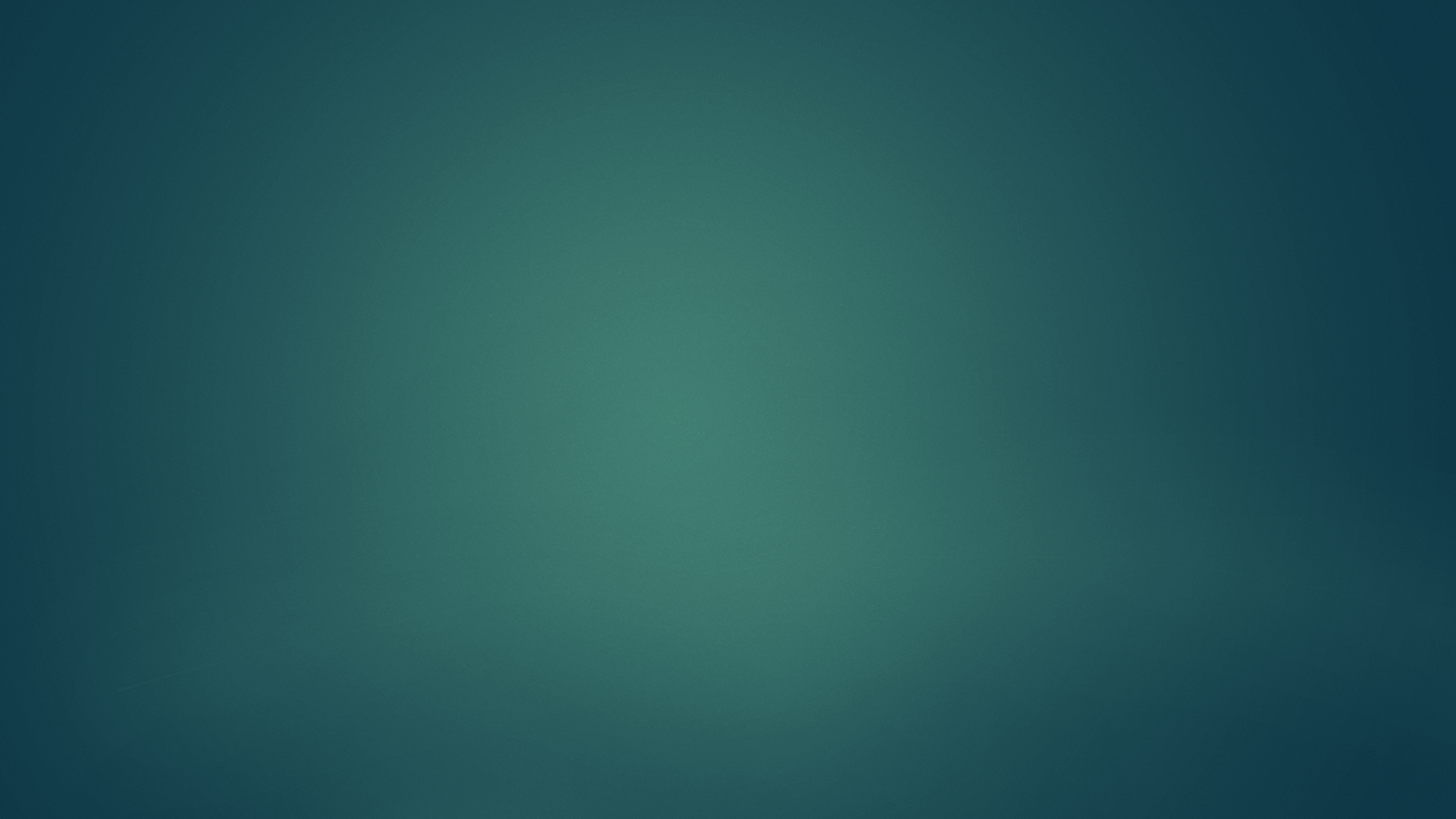

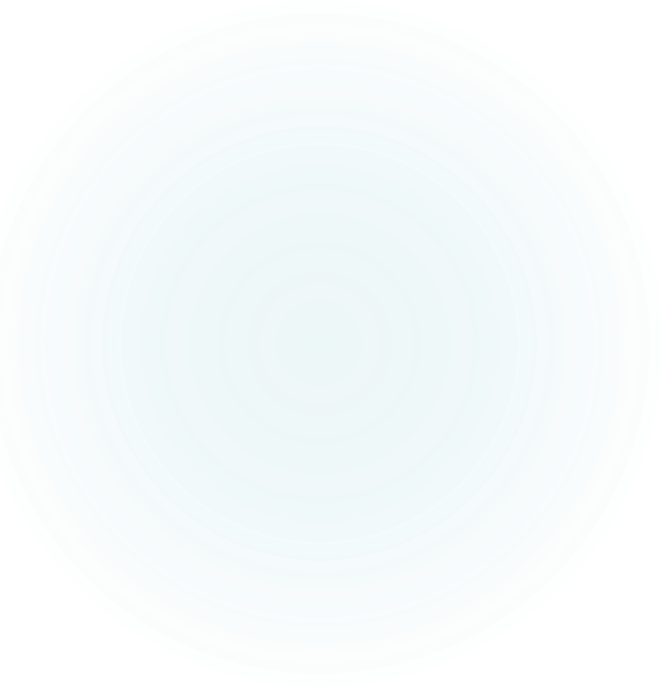

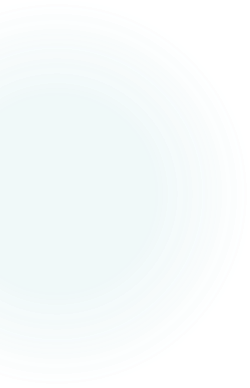

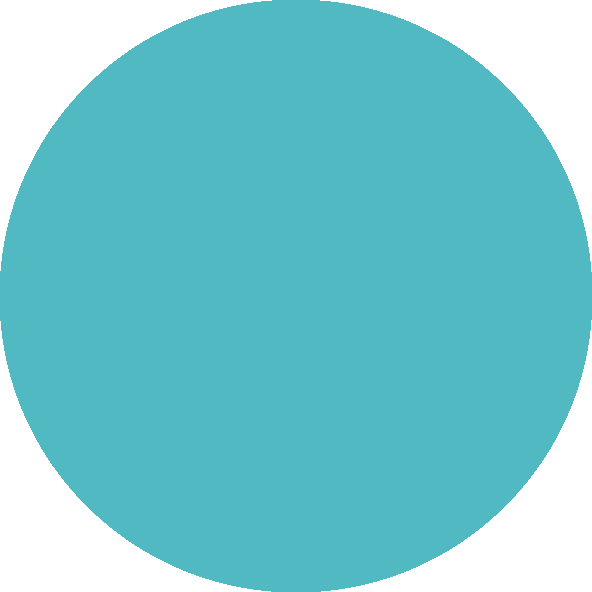

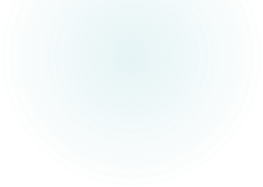

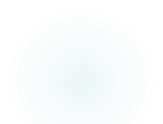

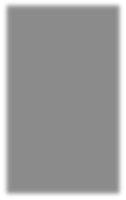


*see notes section for important information regarding each slide contents*

Consider Using biorender.com for making images to add to your slides.

- *SLIDES HAVE BEEN BORROWED (AND EDITED) FROM STUDENT PRESENTATIONS AS EXAMPLES. PLEASE REACH OUT WITH QUESTIONS!*
- *ALONG WITH THE POWERPOINT SLIDES, YOU WILL RECORD A SHORT (3-4 MIN MAX) PRESENTATION TO ACCOMPANY THE SLIDES. THIS CAN BE DONE ON ZOOM.*


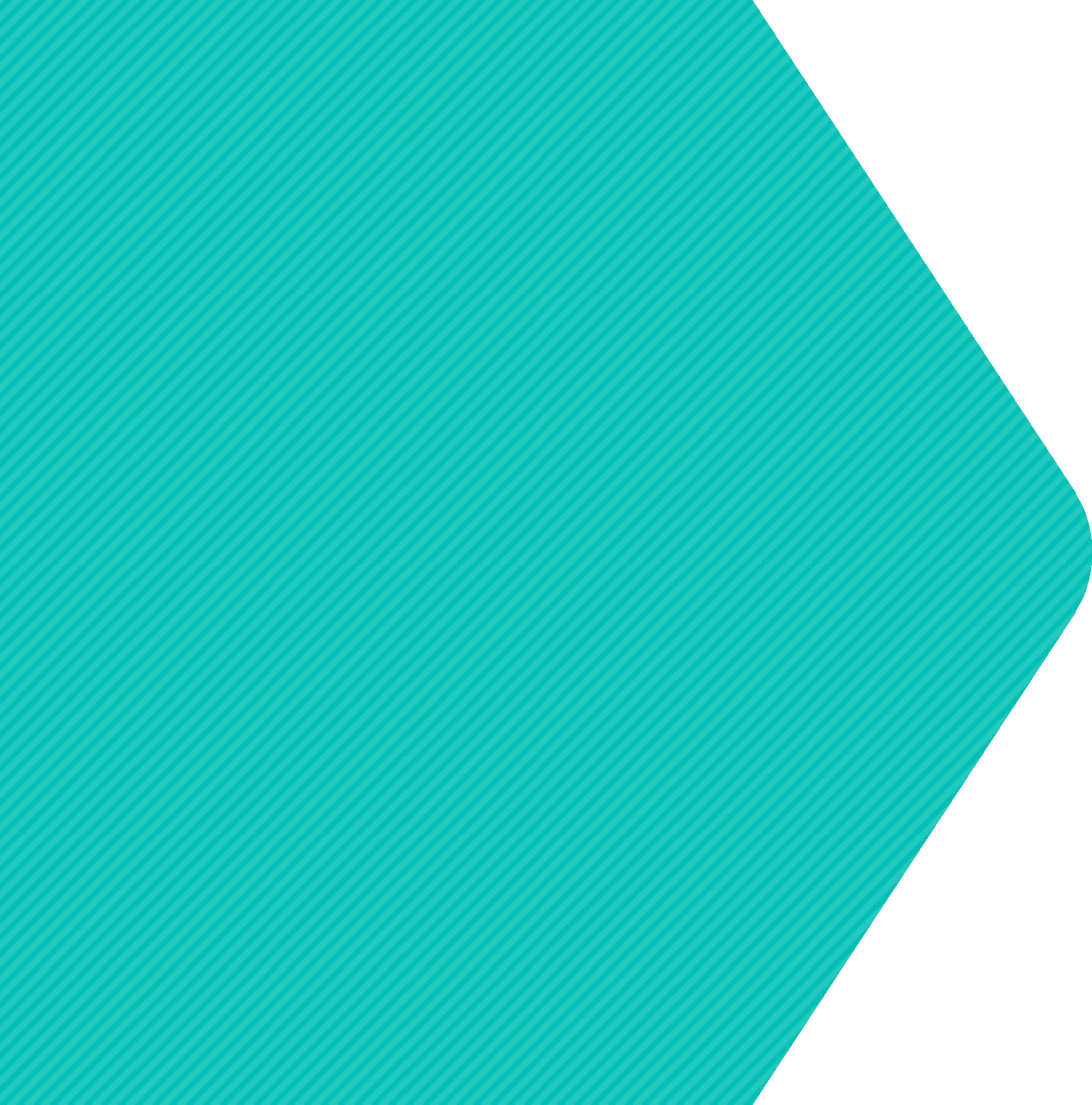

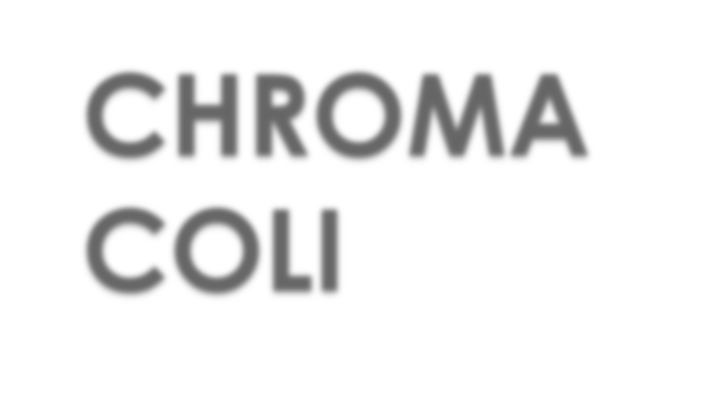

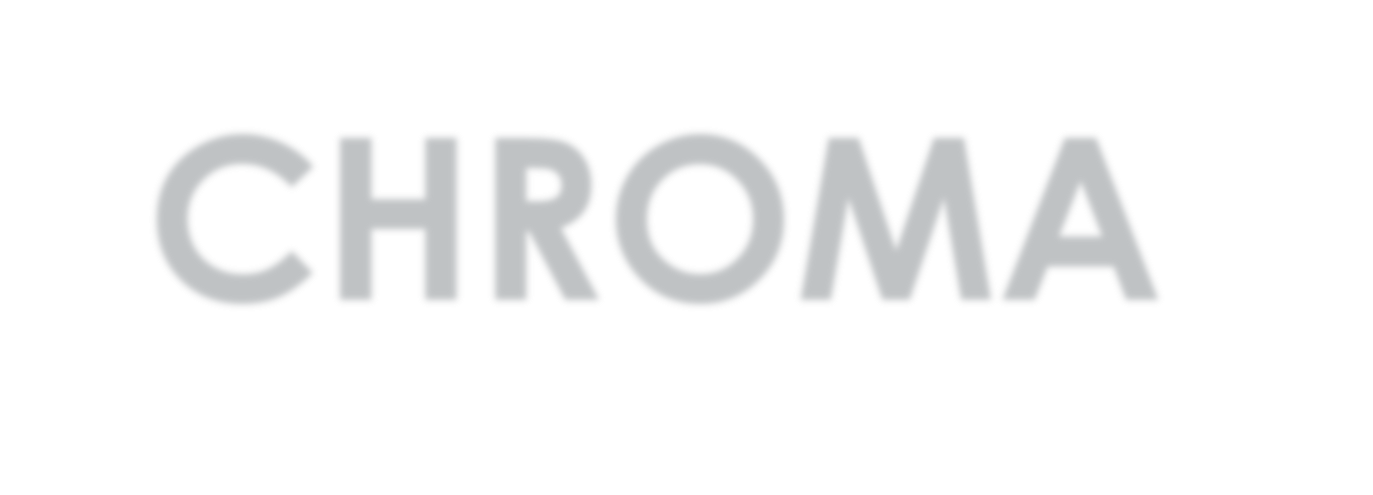

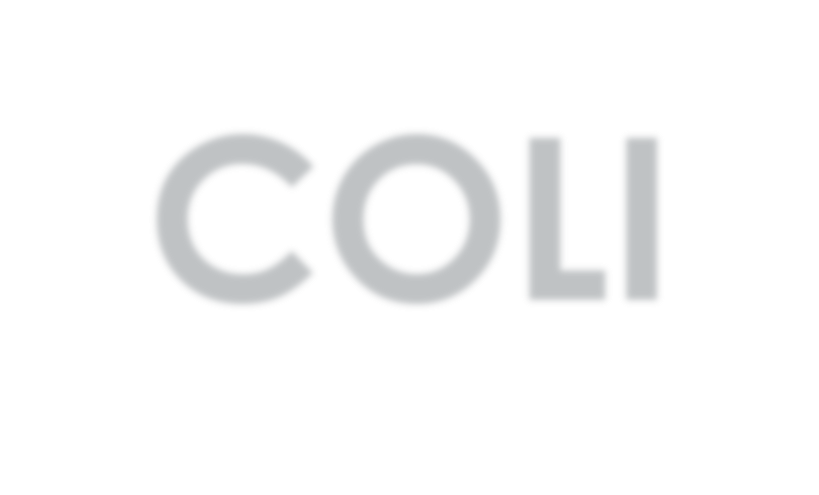

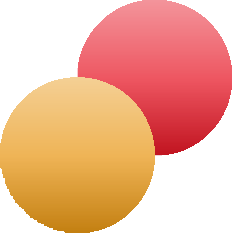

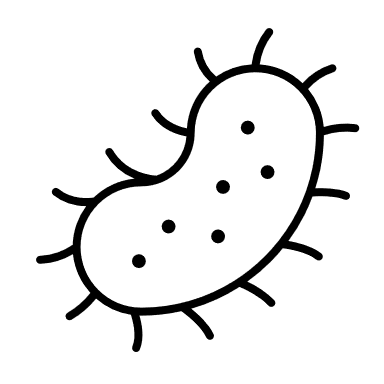

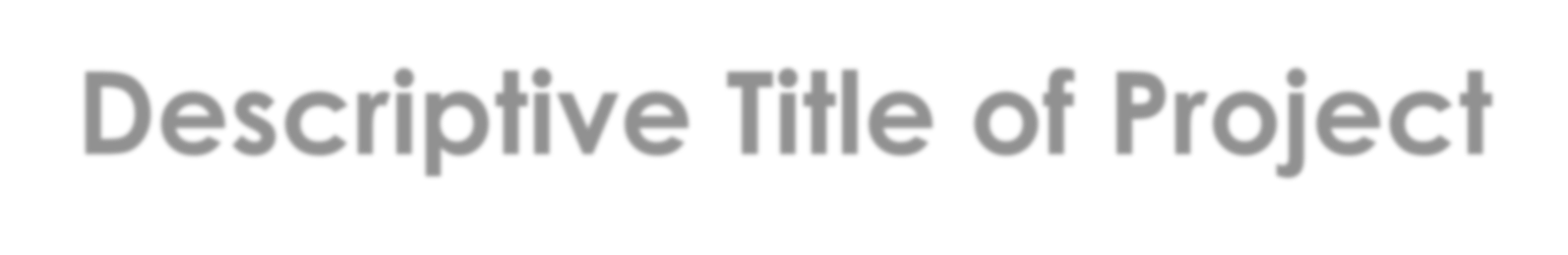


**CHROMA COLI**

**Descriptive Title of Project**

BIOT 777: Molecular Biology and Biotechnology Spring 2021

Student names

Introduction


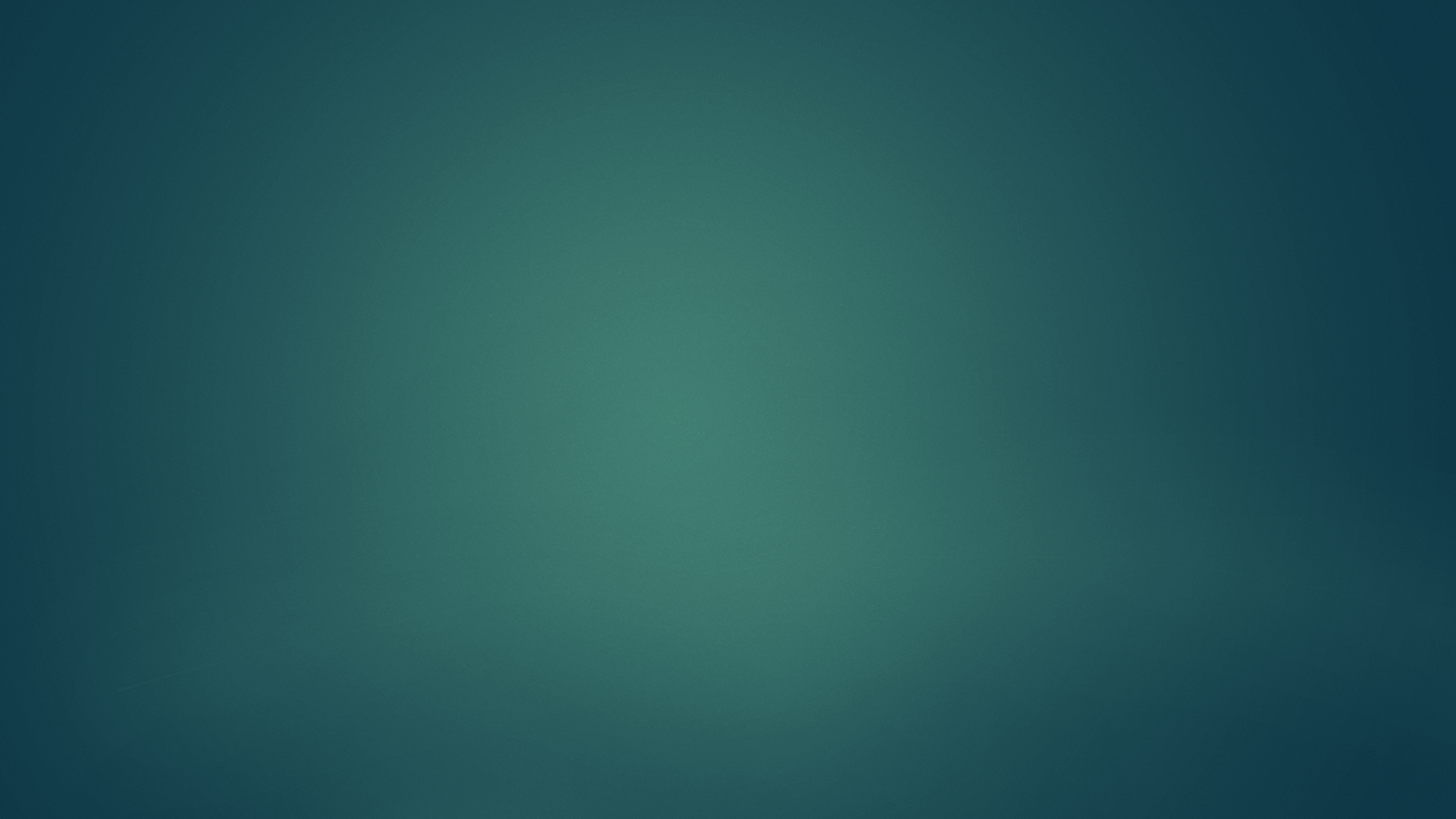

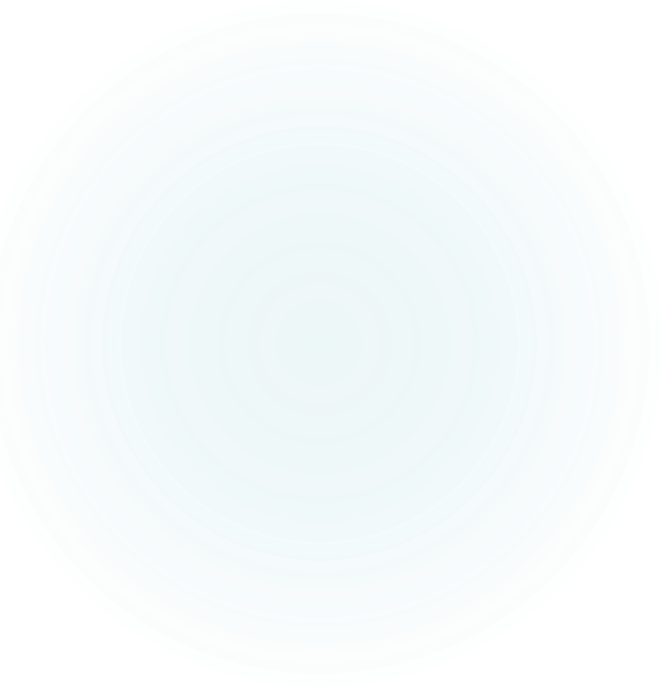

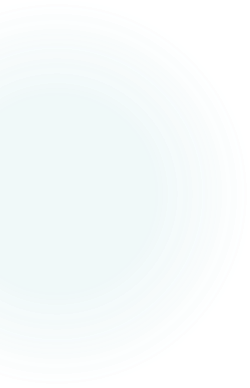

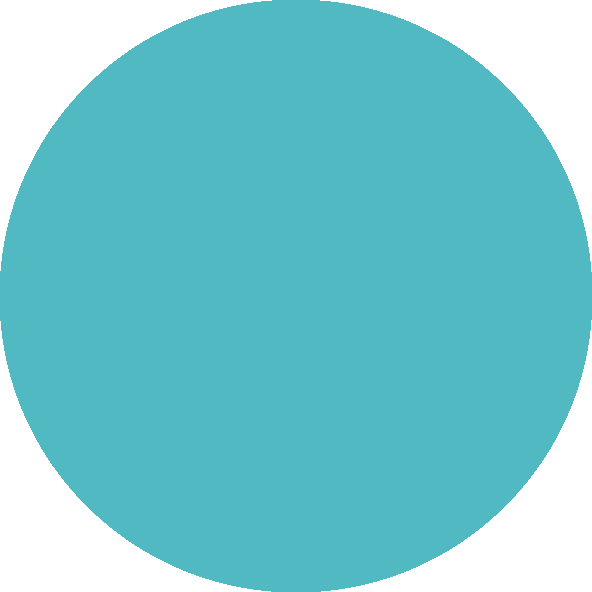

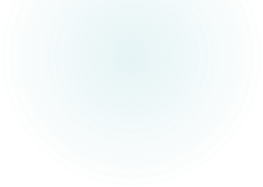

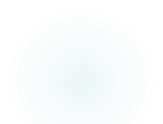

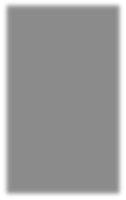

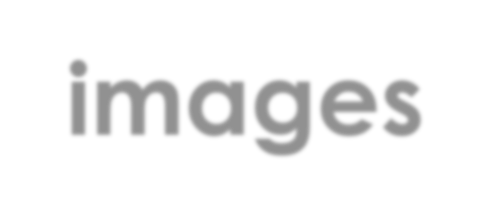

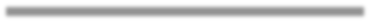


Much of this content can come from the proposal slide(s).

This slide should cover the background of your project/the idea behind the design of your device that you will

present in the coming slides.

Example: If you’ve designed a biosensor for arsenic, why is

this relevant/useful?

Include **images** to represent your ideas rather than just

text.

###### Device Level Design: Black Box Diagram & Truth Table


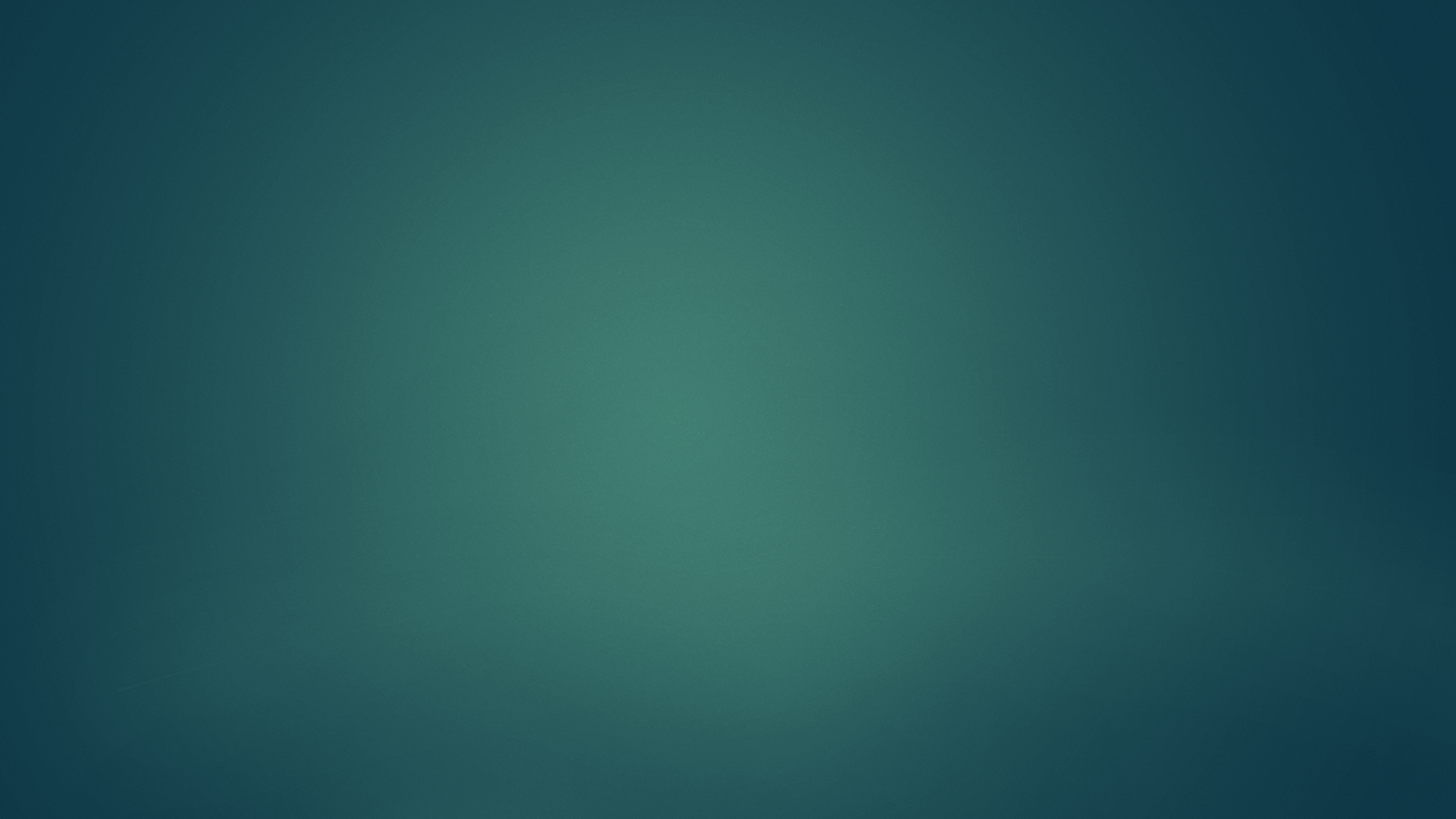

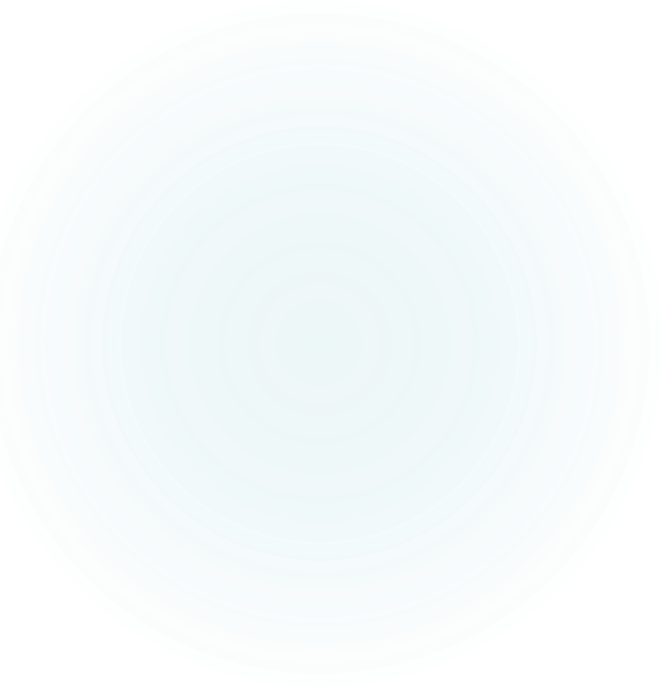

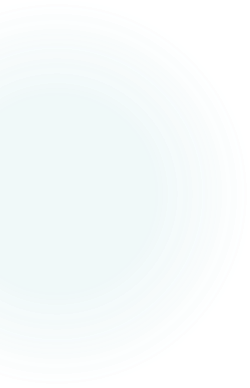

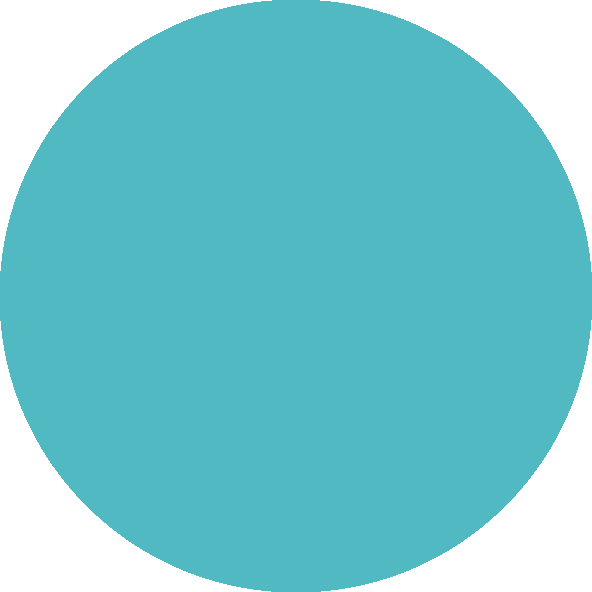

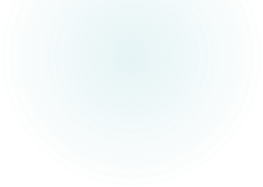

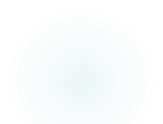

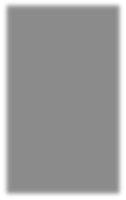

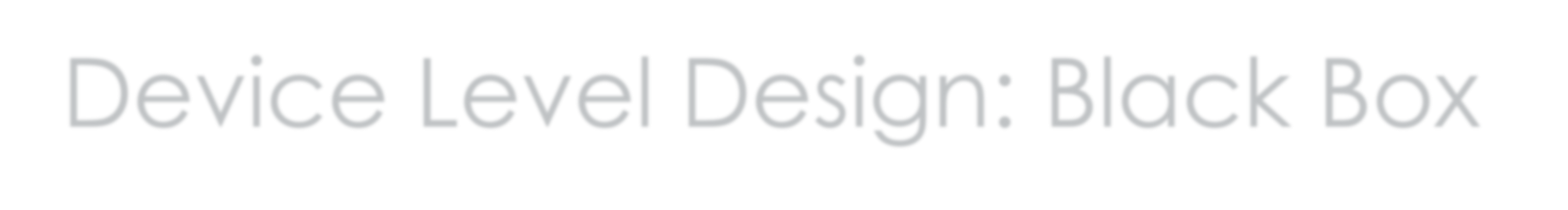

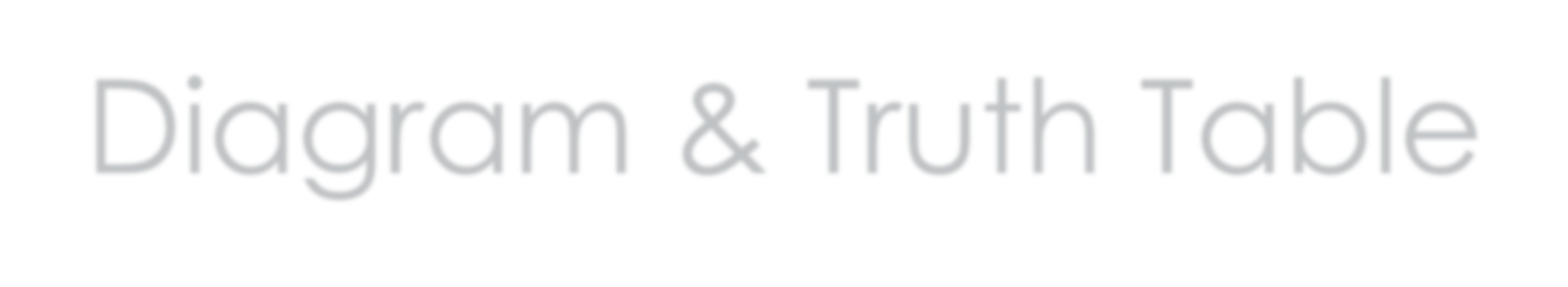

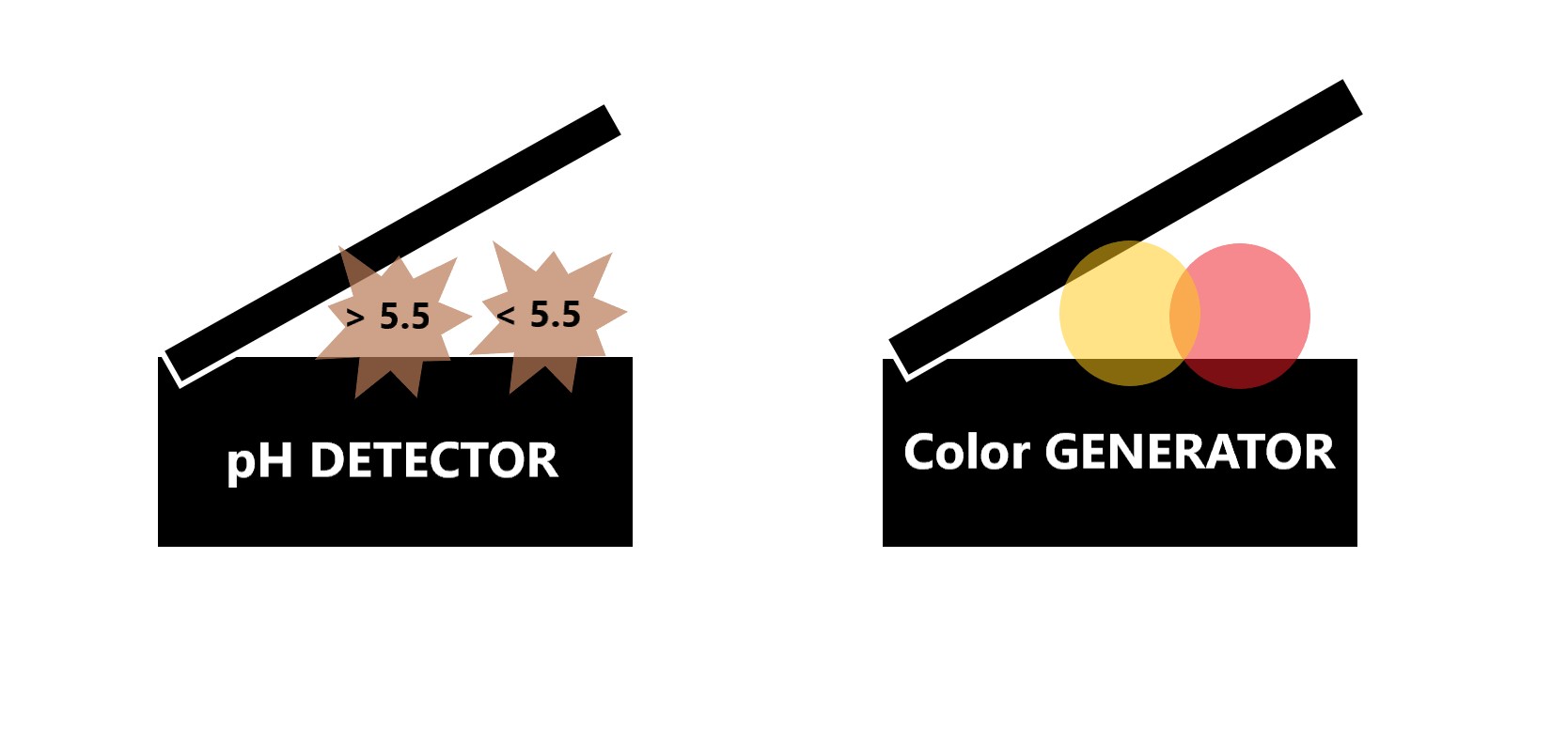


| **Input – pH < 5.5** | **Output – color change red** | **Output – color change yellow** |
| --- | --- | --- |
| + | + | - |
| - | - | + |

Parts Level Design


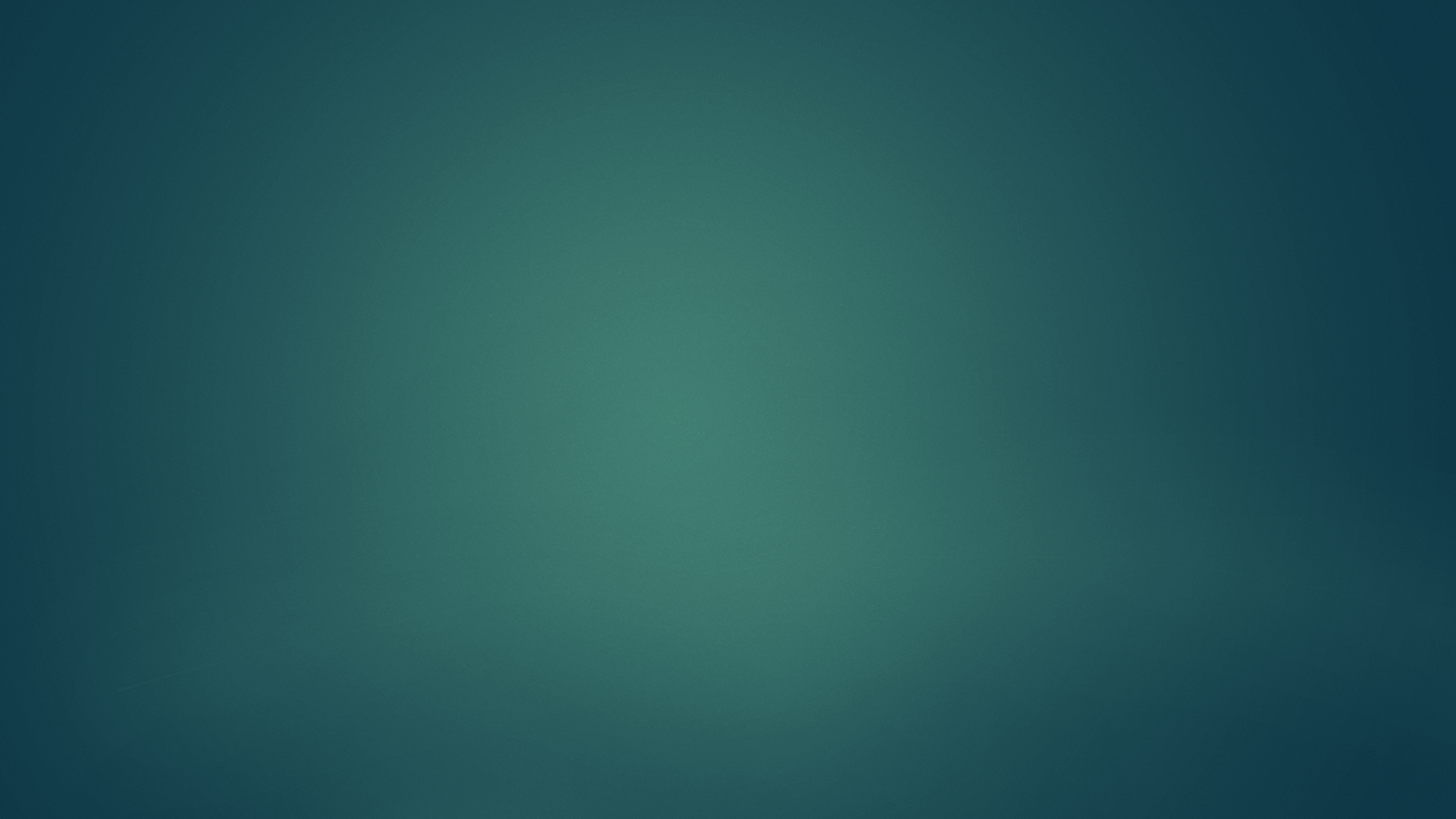

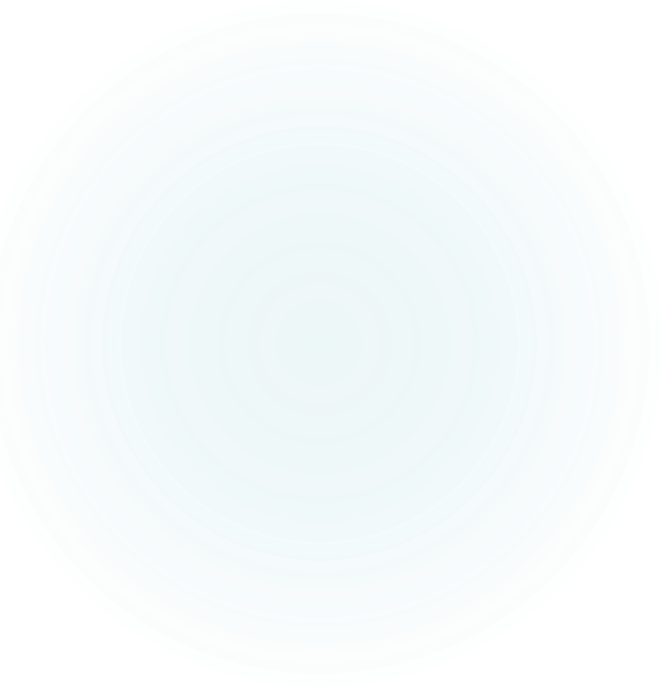

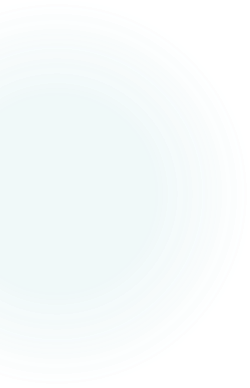

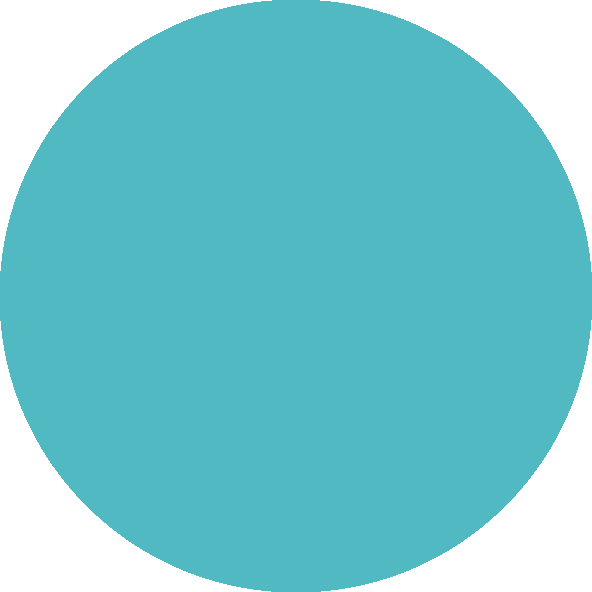

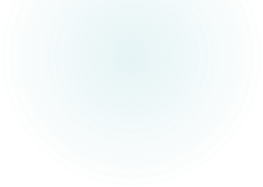

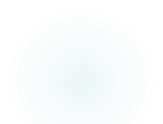

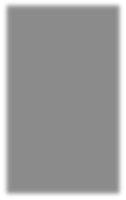

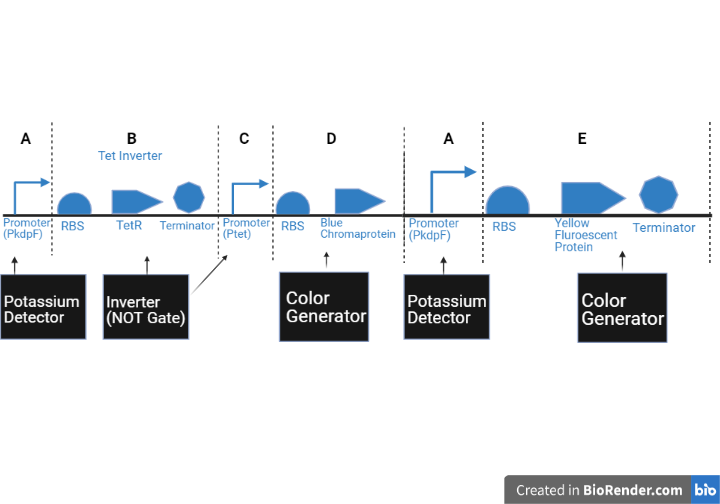


Name/function of parts is given under each device segment.

Note: Segments clearly labeled and divided according to cloning strategy.

Note: Shape of biobrick parts matches iGEM.

| **Part (A-E)** | **Sequence** |
| --- | --- |
| A: BBa_K1682004 | (PkdpF) Potassium Promoter |
| B: BBa_P0440 | Tet Inverter |
| C: BBa_R0040 | Tet Promoter |
| D: BBa_K592025 | Blue Chromoprotein |
| E: BBa_E0430 | Yellow Fluorescent Protein |

Evidence of work

Step 1 – Double Digest

Note: Use cropped gel images!

Step 2 – Ligation

Step 2 – Double Digest

backbone

Call out what is being shown: Yellow fluorescent pellet!

3kb 1kb 500bp

Note: Indicate expected

art sizes. ConPsiadertr A


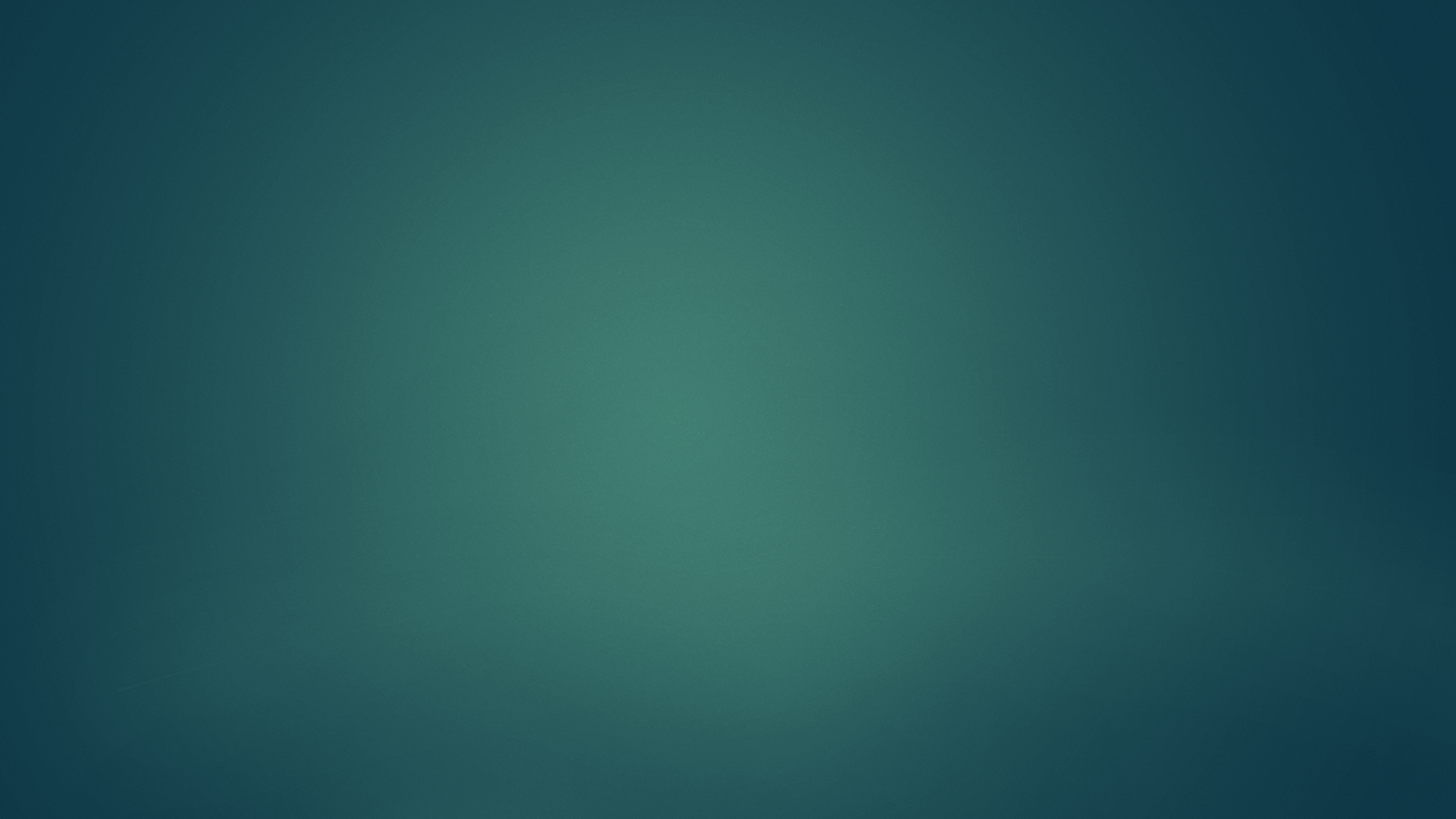

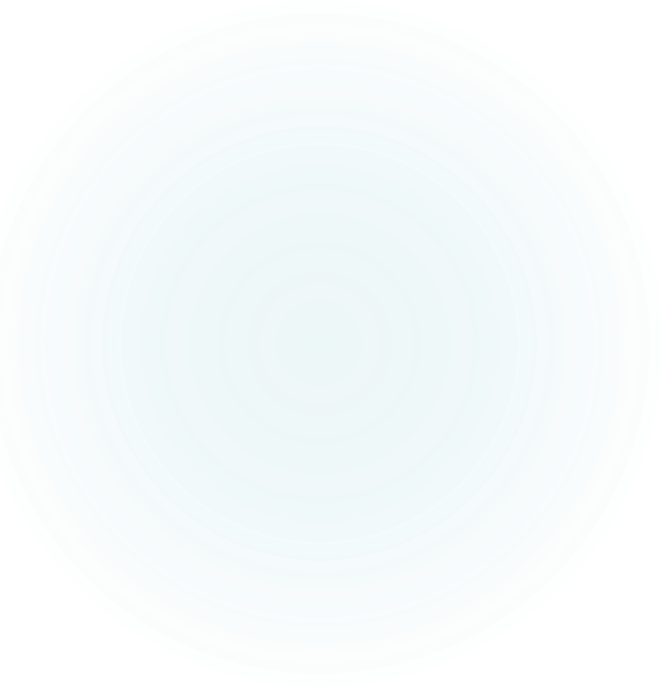

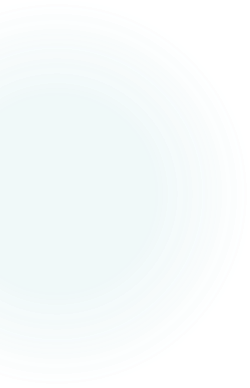

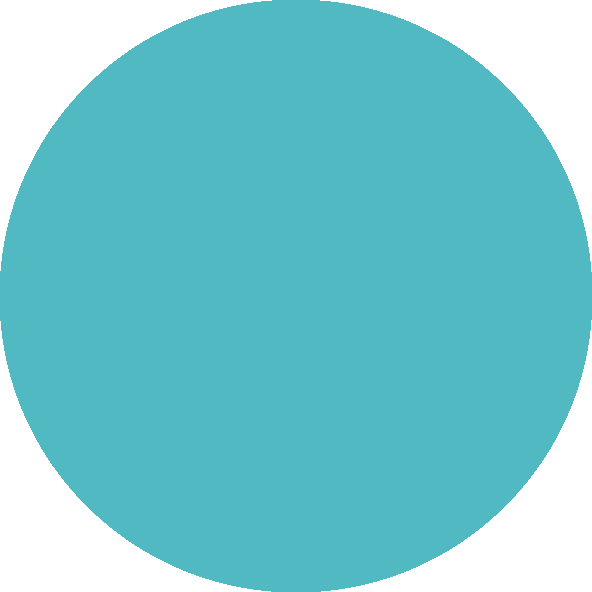

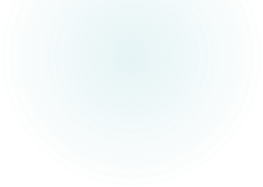

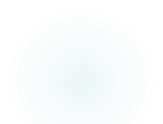

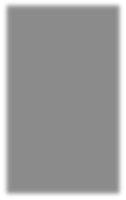

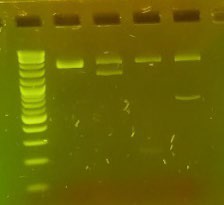

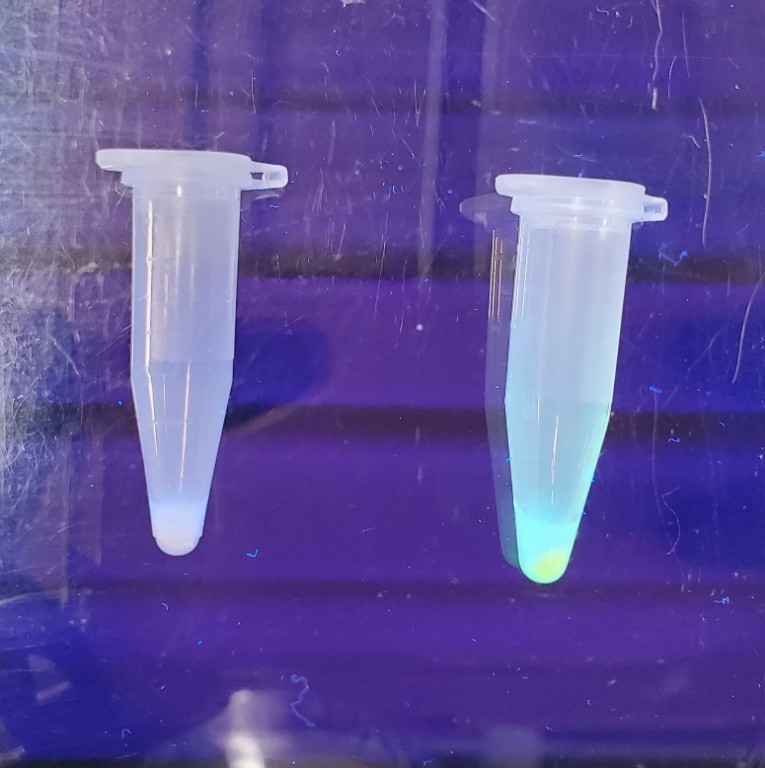

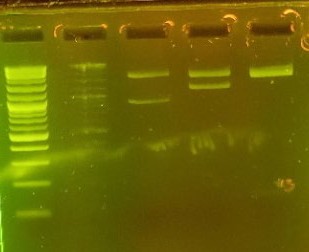


| **Well no** | **Well 1** | **Well 2** | **Well 3** | **Well 4** | **Well 5** | p  **Well 6**u  and |
| --- | --- | --- | --- | --- | --- | --- |
| Part no |  | Ladde r | Part 1 | Part 2 | Part 3 | Part 4 |
| Size |  |  | 54bp | 1307b  p | 220bp | 699bp |

sing arrows (aPbarot v1+ePart2)

/or size indications above.

Part B

(Part 2+Part3 )

| **Well no** | **Well 1** | **Well 2** | **Well 3** | **Well 4** | **Well 5** |
| --- | --- | --- | --- | --- | --- |
| Part no | Ladd er | Ladd er | Part A | Part B | Part 5 |
| Size |  |  | 1.4kb | 951b  p | 1.8kb |

Data/Analysis


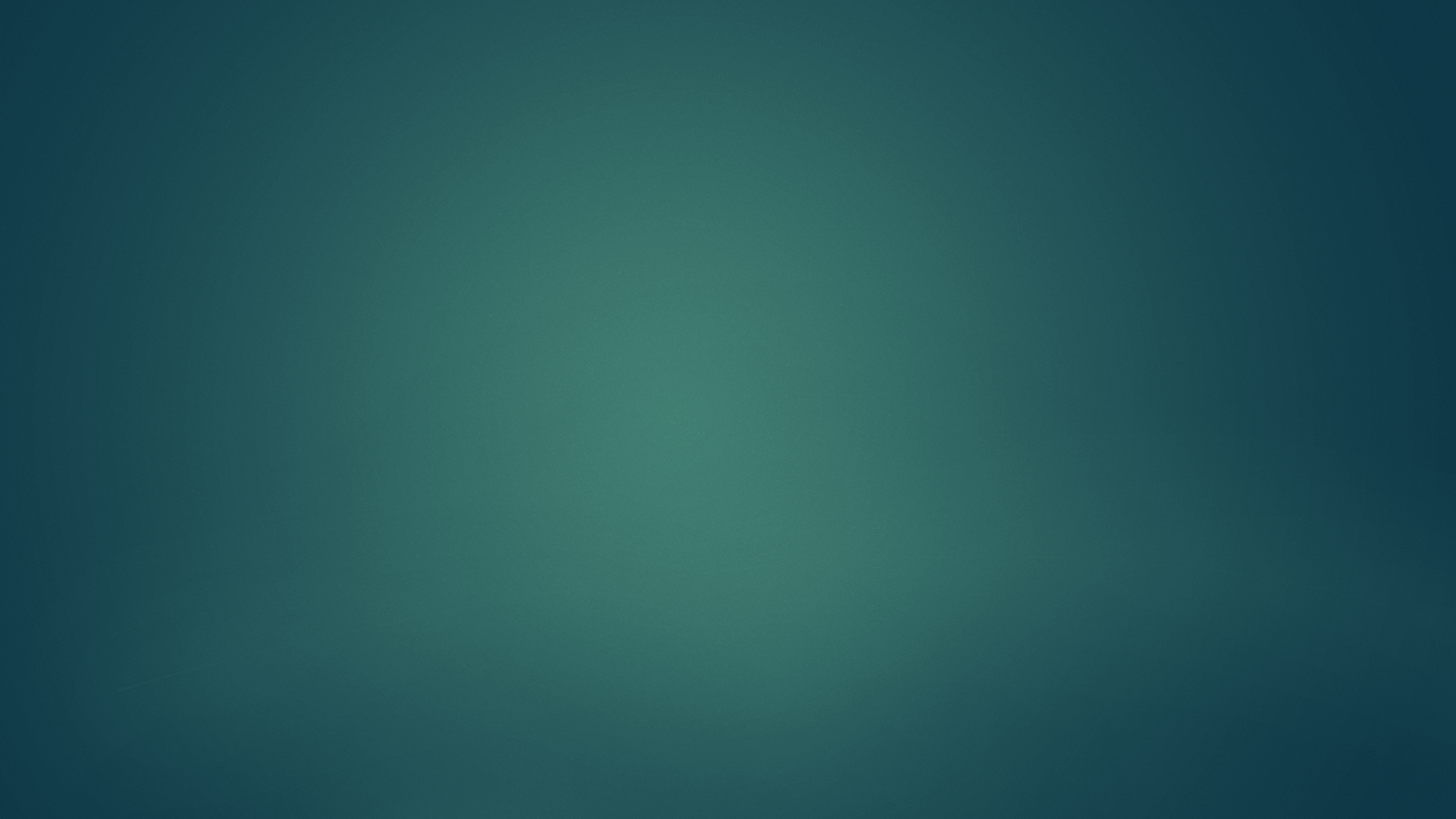

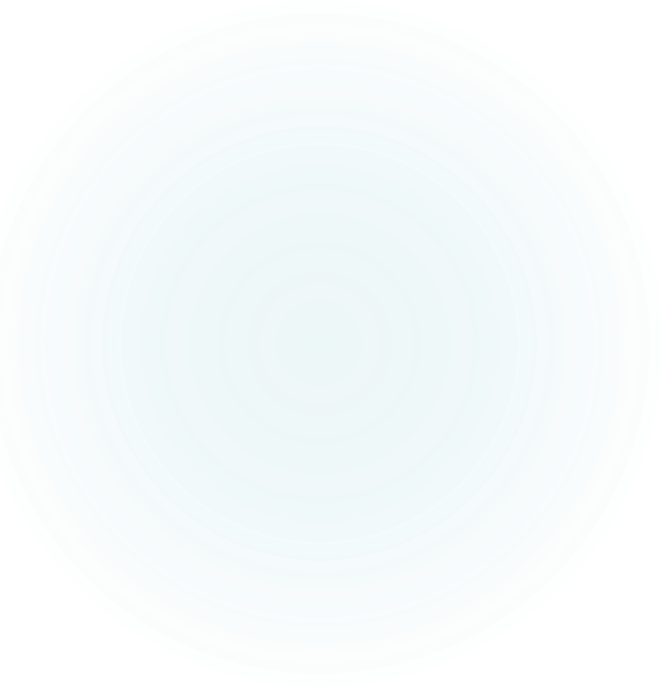

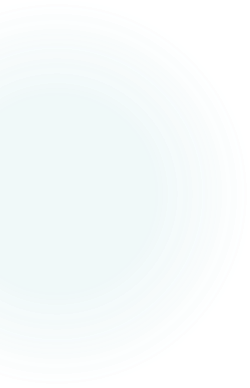

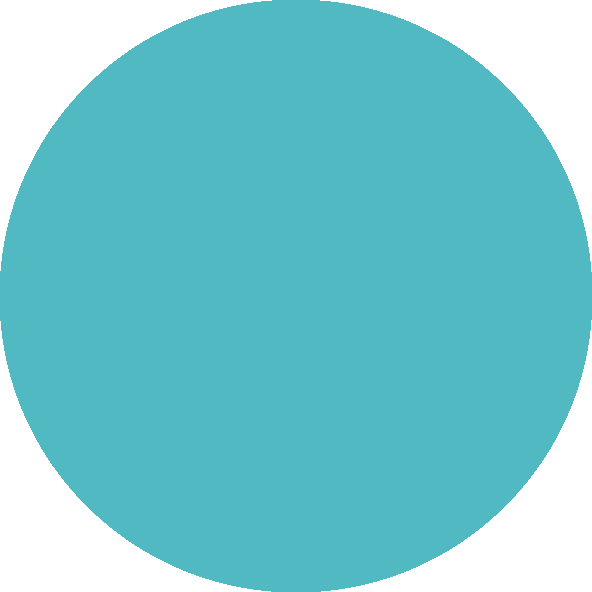

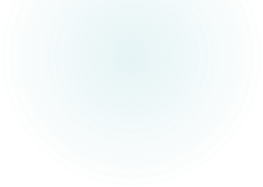

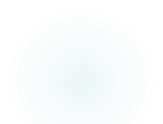

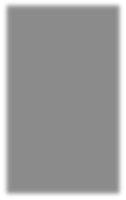

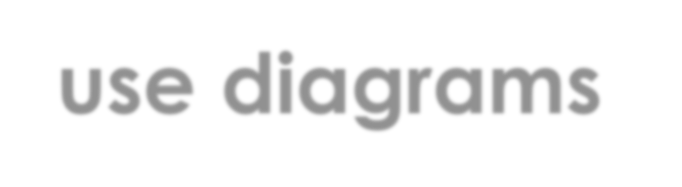

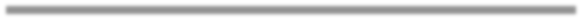

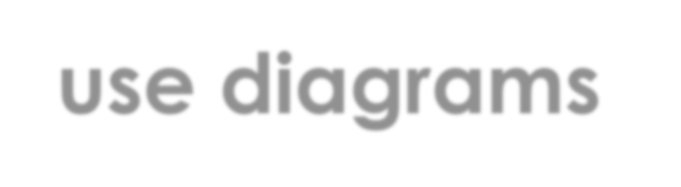

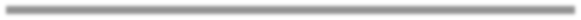


- If you get this far, share what testing of your device you have done (**use diagrams** to display your experimental test design) data you have collected in qualitative or quantitative format(s).
- If you do not get this far, share what precise analysis you *would have carried out* to test your completed device. Be precise here with experimental design/conditions (and **use diagrams** to display your design vs words).

###### Presentation Conclusion


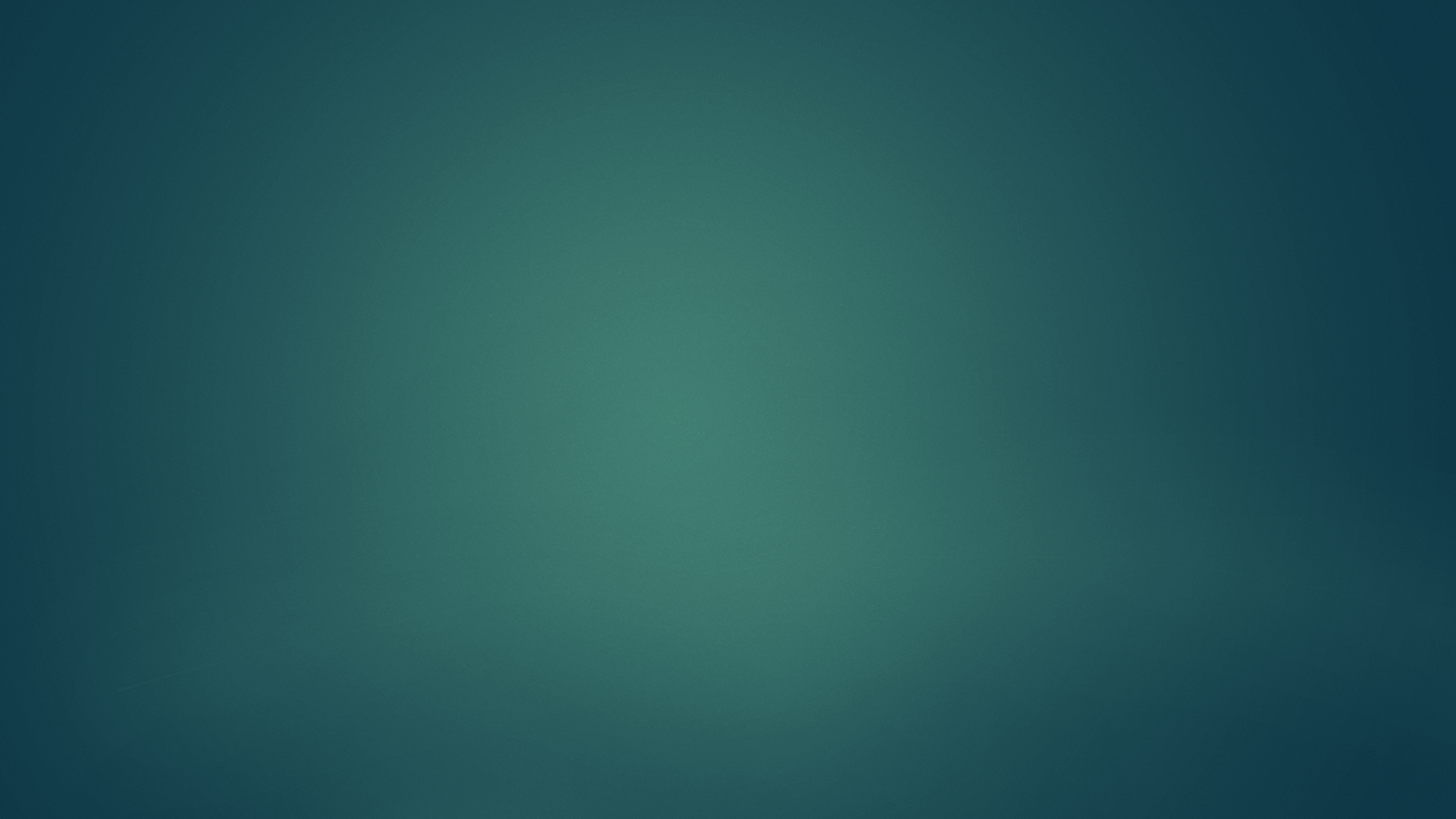

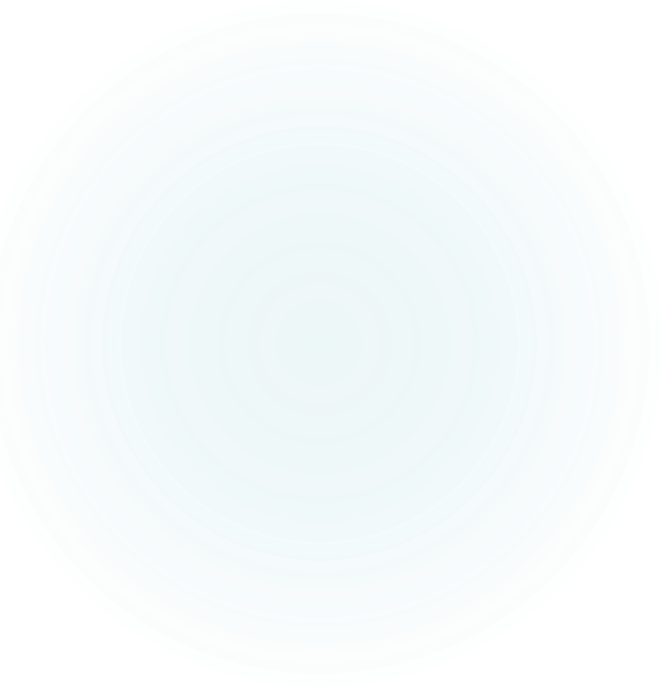

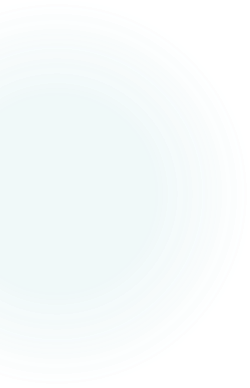

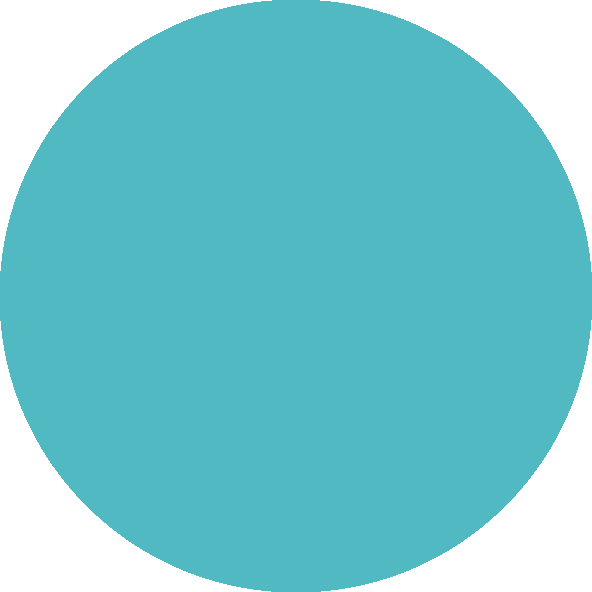

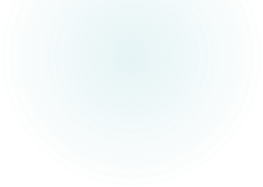

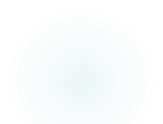

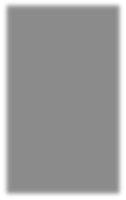


- Wrap up the project presentation with a conclusion statement based upon where you ended your project:
  - Next steps/future directions/problem solving needed/etc.
- Finish the overall oral presentation with…
  - Broad usefulness of Synthetic Biology (can be based upon application of your device design or even more broad)

Device Cloning and Analysis Paper Rubric

**60 pts**.

**Each student** will submit a paper documenting their team’s progress and characterization of their device. These should be written entirely independently, but will draw upon content in your shared Benchling lab notebook and perhaps your shared presentation documents.

Length: **3-5 pages** (**not** including title, name, course, date and literature cited, and diagrams/figures): 12pt font/double spaced

The Lab Paper Must Include the Following Sections. In order to be clear, the sections may be headed with titles and letters corresponding to the information below. Example: “Abstract”; “Section 1.b”

**Abstract:** *Brief starting summary paragraph of your team’s overarching goals, methods, and achievements with your research.*

Section 1: **Device Description and Cloning**

1. Figure showing final (original) design for orientation of parts. This should be well annotated with part numbers and part functions.
2. Detailed table of original parts used (part numbers, size, backbone)
3. A **concise description** of your step-by-step cloning strategies and a flow chart showing cloning steps.
4. Gel images proving completed (or partially completed) device *(each well must be labeled and each band clearly identified; also must include Figure captions)*
5. **If you did not complete your device***:* Be clear as to what step(s) you stopped at in the process; what is known and what may be unclear.
   1. What are the steps that still needed to complete the device to get it to be functional?
   2. If there are unclear aspects to the cloning process (e.g., something was not working when you stopped your cloning experiments), you **must** offer rational hypotheses as to WHY.

Section 2: **Device Analysis and Results**

1. Detailed proposal for analysis, including *(as it applies to your project):*
   1. concentrations and ranges for inducers, testing occurring in liquid or solid media, length of time for testing, how the reporter (color/fluorescence) was going to be assessed (quantitatively or qualitatively)
2. Describe any/all methods used to analyze that your device functioned as designed ***(only applies to those that were able to bring their device to a functional status****); include appropriate, well-labeled figures to demonstrate device function*
3. Research the use of at least one of the parts (preferably, something like an inducible

promoter) in iGEM and/or primary literature, and write a section summarizing how others have utilized this part in functional devices in the past.

- 1. What type of device was engineered? How have they tested its function? Under what specific conditions did the device turn on/off?
  2. How might this information help explain why your device may not have worked under the specific conditions you tested (if your project progressed this far)?

**Grading Rubric**

| **Section** |  |
| --- | --- |
| **Abstract** | *10 pts* |
| **Section 1** | *30 pts* |
| **Section 2** | *20 pts* |


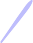

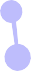

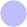

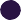

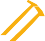

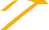

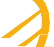

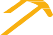

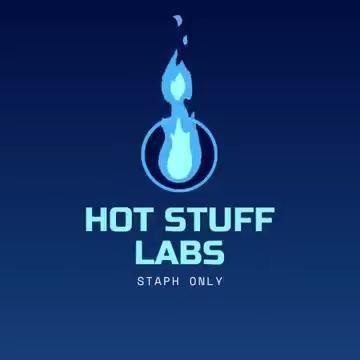


Hot Stuﬀ’s Project

Proposal

Introduction

Staphylococcus aureus is an opportunistic pathogen that invades many tissue types and causes infection most commonly in the skin, but is known to travel through the bloodstream to infect bone and heart valves. Many strains of S.aureus are resistant to antibiotics, namely methicillin-resistant staphylococcus aureus (MRSA), a prevalent nosocomial infection.

In high heat (physiological fever temperatures, above 35 C) our device will begin to shed its repressor and transcribe the engineered coding sequence: a gene for the enzyme lysostaphin


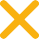
The cellular response of our engineered cell will be to produce and secrete the mature lysostaphin enzyme and eﬀectively lyse *S.aureus*.

Systems Level Design

Chassis: E. coli DH5alpha for final testing

Truth Table

| Heat (>30℃) | Lysostaphin Production |
| --- | --- |
| 0 | 0 |
| 1 | 1 |

Device Level Design: Black Box

Abstraction

Device Level Design: Wiring Diagram

Logic Device

Parts Level Design Part 1

Parts Level Design Part 2

Expected Results

- In temperatures above 35C we expect to see clear zones of inhibition of S. aureus around the regions of our engineered E. Coli.

- This will result will confirm the lysis of S. aureus due to the successful production and secretion of the lysostaphin enzymes from our engineered E. Coli

- As the temperature increases we expect that the zones of inhibition will also increase in size
- When the temperature is below 30C we expect to see no clear zones of inhibition around the regions of our engineered E.Coli

Analysis/Tests

- Will be testing against *S. auerus* on solid LB media in varying amounts of temperatures.
- Streaking the entire plate with *S.*

*auerus* and streaking small, labelled regions of our engineered E.Coli on top.

*S. aureus* lawn

*E. coli (*w/ device)

- This plate will be incubated overnight at a

range of temperatures and compared against a plate of *S. aureus* with wild type *E. coli* incubated under the same conditions.

- We would measure the zone of inhibition created at each separate temperature as a marker for system eﬀectiveness.

Taken from: http://parts.igem.org/Part:BBa_K748002

26℃, 30℃, 37℃, 40℃, 42℃, 45℃, 48℃ testing temperatures

Potential Problems

- - Incubating plates in high temperatures may cause deformation of the agar and make it difficult to analyze data
    - Solution: Limiting our testing maximum to 48℃ (which is well below the melting point of our agar) will help with this potential issue.
  - We do not have many incubators to run all our temperature tests simultaneously
    - Solution: Run tests using 1-2 incubators at a time. It may take longer to collect data, but it is feasible if we have time. If we are short on time, we can cut out the extrema our temperature tests and focus on the middle range of temperatures to analyze data.
  - The only way we would know if our device is working properly would be that it kills S.aureus. If this does not occur, the cause would be unclear. It may be that our device is non-functional, or it may be because of an external factor.
    - Solution: If this situation occurs, we can add a RFP to our device which will help us know of our device is working or not.

Table of Parts

| Part ID | | Name | Size | Well Location | 2021 Year Plate | Plasmid Backbone | Type | Description |
| --- | --- | --- | --- | --- | --- | --- | --- | --- |
| BBa_k608351 | | Temperature Sensitive Promoter | 948bp | 6N | 2021 Plate 1 | pSB1C3 | Promoter | Consists of a constitutive promoter, temperature sensitive repressor protein, and a cl regulated promoter |
| BBa_S05050 | | RBS and Lysostaphin ORF | 759bp | 5B | 2021 Plate 1 | pSB1C3 | RBS and ORF | Composed of an RBS and a lysostaphin coding sequence that produce enzymes to kill S. aureas |
| BBa_B0015 | | Double Terminator | 129bp | 4E | 2021 Plate 3 | pSB1C3 | Terminator | Terminates transcription |
|  |  |  |  |  |  |  |  |  |
|  |  | | | | | | | |

Bibliography

*Digital buffer and the TRI-STATE Buffer Tutorial*. Basic Electronics Tutorials. (2018, October 9). Retrieved September 19, 2021, from [ht tps:// www.electronics-tutorials.ws/logic/logic_9.html](https://www.electronics-tutorials.ws/logic/logic_9.html).

Qiao, L. (2012, September 15). *Truncated lysostaphin coding sequence. Lysostaphin has has a specific lytic action against S.aureus.* Registry of Standard Biological Parts. Retrieved September 19, 2021, from http://parts.igem.org/Part:BBa_K748002.

#### The Operana

Project update

[This Photo](https://laescaleradeiakob.blogspot.com/2015/01/los-platanos-de-silo.html) by Unknown Author is licensed under [CC BY-SA-NC](https://creativecommons.org/licenses/by-nc-sa/3.0/)

Parts Level Design

R0040 P0312

J04500 K592010

J45219

(Lac I)

(Amil GFP)

(ATF1)

RBS

ORF

RBS

ORF

RBS

ORF

Step 1 : Part 1

Part 2

Part 3

Part 4 Part 5

Step 2 :

Step 3 :

Part 1 +Part 2 = Part A Part 3 + Part 4 = Part B Part 5

Part A + Part B = Part X Part 5 = Part Y

Step 4 :

Part X + Part Y = The Operana Bacterium (Indole deficient chassis)

Evidence of work

Step 1 – Double Digest

Step 2 – Ligation

Step 2 – Double Digest

Part A

| **Well no** | **Well 1** | **Well 2** | **Well 3** | **Well 4** | **Well 5** | **Well 6** |
| --- | --- | --- | --- | --- | --- | --- |
| Part no |  | Ladder | Part 1 | Part 2 | Part 3 | Part 4 |
| Size |  |  | 54bp | 1307bp | 220bp | 699bp |

(Part 1+Part2)

Part B

(Part 2+Part3 )

| **Well no** | **Well 1** | **Well 2** | **Well 3** | **Well 4** | **Well 5** |
| --- | --- | --- | --- | --- | --- |
| Part no | Ladder | Ladder | Part A | Part B | Part 5 |
| Size |  |  | 1.4kb | 951bp | 1.8kb |

Next steps

Final Output

1. Complete Step 3 and Step 4
2. Final device – LB broth and TSA plate
3. Perform the analysis and confirmatory tests for color and smell
   - - - Testing color:

-Primary visual observations

-Bradford assay and linear regression

- - - - Testing the banana smell:

-Primary observations

-Banana extract oil (standards in different concentrations + a negative control)

Created with BioRender.com

Problems Encountered

|  | **Part 5 (Bba_J45219)** | **DNA Conc** |  |
| --- | --- | --- | --- |
|  | 1^st^ extraction | 3.99 ng/uL |  |
|  | 2^nd^ extraction | 22.2 ng/uL |  |
|  | 3^rd^ extraction (newly constructed) | 90.1 ng/uL |  |

1. Part 5 (BBa_J45219) had to be newly constructed, due to **low DNA concentration**

1. Part 3 (Bba_J04500) had to be re-extracted due to **pipetting mistake** that used the whole volume for digestion.
2. Problem with gel 1:

Parts A, B, and 5 were re-digested – **Digestions didn’t work OR problem with the gel.**

1. Problem with gel 2:

Part B band doesn’t match the virtual digest –

**Digestion didn’t work OR wells were switched.**

1. Problem with gel 3: OMG!

Gel 1 Gel 2

**How does time affect GFP and Chromo Blue with a**

**Medium Promoter**

Parts Used

- Medium Promoter + Weak RBS (BBa_K608007; 57 bps)
- **GFP** (BBa_E oo4o; 720 bps)
- **Chromo Blue** (BBa_K 592009; 669 bps)
- **Strong Promoter** + Weak RBS (BBa_K 608004; 57 bps)

Project Execution

1. Grow all individual parts
2. Ligate Chromo Blue to Medium Promoter + RBS
3. Ligate GFP to Medium Promoter + RBS
4. Ligate GFP or Chromo Blue to Strong Promoter + RBS (if time permitted)
5. Test presence of ligated parts with a gel
6. Make streak plates of ligated parts and take pictures over time to see when GFP starts looking green, and when Chromo Blue starts looking blue

Parts and Concentrations

The picture can't be displayed.

GFP Pick 2:

Con.= 55.8 ng/uL

260/280= 1.86

260/230= 2.31

Med. Prom. Pick 1:

Con.= 31.1 ng/uL

260/280= 1.96

260/230= 2.20

Chromo Blue Pick 3:

Con.= 35.6 ng/uL

260/280= 1.32

260/230= 1.44

Strong Prom Pick 1:

Con.= 10.4 ng/uL

260/280= 1.81

260/230=1.59

Digestion

E X-Part-S

P

E X-GFP-S

P

E -X-S- P

E-X-Part-M-GFP-S-P

DNA Digestion used for Gel

There is a faint line where we expected to see Chromo Blue and a line where we expected to see GFP. The Strong promoter and Medium Promoter were not able to be seen since they show up around 57 bps.

Ligation of Parts

Used Kanam. Backbone and had contamination

Used Kanam. Backbone and had contamination

Used Tetra. backbone after Kanam. was found to have contamination. No growth.

Next Steps with Ligations

- Made broth from picks from Ligation plates of Med. Promoter+GFP and Med. Promoter+ Chromo Blue. Made streak plates using broth from ligation plates. (We decided to abandon the Strong Promoter+Chromo Blue given time constraints. )
- Extracted DNA, determined DNA concentrations from Nanodrop, and digested the ligation using ECOR1 and PST1
- Ran a gel with ligated, digested parts.

Ligated Parts

GFP+Medium Promoter 24 hrs. of growth

No Blue light

GFP+Medium Promoter 24 hrs. of growth Under Blue Light

Chromo Blue+Medium Promoter

48 hrs. of growth

3:00 pm

Day 1 results show GFP beginning to fluoresce after 12 hours. Chromo Blue and Killer Red results show growth but Chromo Blue is not showing much color yet.

The picture can't be displayed.

Day 2 6:00pm

Day 2 9:00pm

Day 2 results show Chromo Blue is visibly light blue after 24 hours, but a darker blue color develops over time. GFP does not appear any brighter after 30 hours of incubation.

Day 2 9:00pm

Day 3 9:00am Day 3 9:00am

Day 3 showed no new results. It can be determined by our pictures that Chromo Blue takes 36 hours to reach the deepest color blue it can be. After 30 hours of incubation, GFP does not get any brighter.

GFP begins fluorescing after 12 hours and Chromo Blue looks blue after 24 hours.

**Questions?**
